# Measurement of Fecal Energy Loss Using Bomb Calorimetry in Adult Patient Populations: A Systematic Review and Meta-Analysis

**DOI:** 10.3390/nu18182976

**Published:** 2026-09-11

**Authors:** Maridi Aerts, Eline Verheyen, Lynn Leemans, Hendrik Reynaert, Elisabeth De Waele

**Affiliations:** 1 Metabolism and Nutrition (MENU) Research Unit, Faculty of Medicine and Pharmacy, Brussels Health Campus, Vrije Universiteit Brussel (VUB), Laarbeeklaan 103, 1090 Brussels, Belgium; eline.verheyen@uzbrussel.be (E.V.); lynn.leemans@uzbrussel.be (L.L.); elisabeth.dewaele@uzbrussel.be (E.D.W.); 2 Department of Gastroenterology, Brussels Health Campus, Universitair Ziekenhuis Brussel (UZ Brussel), Laarbeeklaan 101, 1090 Brussels, Belgium; hendrik.reynaert@uzbrussel.be; 3 Department of Clinical Nutrition, Brussels Health Campus, Universitair Ziekenhuis Brussel (UZ Brussel), Laarbeeklaan 101, 1090 Brussels, Belgium; 4 Vitality Research Group, Faculty of Physical Education and Physiotherapy, Brussels Health Campus, Vrije Universiteit Brussel (VUB), Laarbeeklaan 103, 1090 Brussels, Belgium

**Keywords:** stool bomb calorimetry, fecal energy loss, intestinal energy absorption, energy malabsorption, nutritional adequacy, gastrointestinal dysfunction, systematic review, meta-analysis

## Abstract

**Background/Objectives:** Accurate assessment of nutritional adequacy requires insight not only into energy intake but also into the proportion of nutrients absorbed. Stool bomb calorimetry quantifies fecal energy content and estimates intestinal energy absorption, but its application and reporting vary. We aimed to evaluate its application in adult patient populations and to quantify intestinal energy absorption and its variability. **Methods:** PubMed, Embase, ClinicalTrials.gov, BASE and NDLTD were searched without date restriction (PROSPERO CRD420261389214). Two reviewers screened independently, and risk of bias was assessed using design-appropriate JBI checklists. Studies reporting percentage absorption with recoverable variance were pooled using a random-effects model; a post hoc subgroup analysis by patient population was performed. **Results:** Twenty-eight studies (1981–2026) were included; seventeen contributed to the meta-analysis (439 participants). Absorption ranged from 50.0% to 91.9%. The pooled estimate was 74.2% (95% CI 67.9–80.5), with substantial heterogeneity (I2 = 96.3%) and a 95% prediction interval of 48.8–99.6%. Patient population explained most of the between-study variance (pseudo-R2 = 82%): 62.9% (95% CI 57.4–68.5) in short bowel syndrome and intestinal failure versus 85.1% (95% CI 81.2–88.9) in other populations (*p* < 0.001). Calibration and replicate determinations were each reported in one of 28 studies. **Conclusions:** Intestinal energy absorption varies widely across adult patients, but the variation is largely structured by population. Patients with short bowel syndrome or intestinal failure absorb approximately 63% of ingested energy, more than 20 percentage points less than other adult populations. Prescribed energy intake should not be assumed to equal absorbed energy. Standardized reporting of measurement procedures is needed before clinical use can be evaluated.

## 1. Introduction

Accurate assessment of nutritional adequacy requires insight not only into prescribed or delivered energy intake, but also into the proportion of nutrients that is effectively absorbed by the gastrointestinal tract [1,2]. In adult patient populations, impaired digestion or absorption may lead to clinically relevant energy losses, resulting in hidden caloric deficits despite apparently adequate nutritional provision [1,2].

Indirect calorimetry (IC) is increasingly used in clinical and research settings to obtain patient-specific measurements of resting energy expenditure (REE) and is considered superior to predictive equations in metabolically unstable or heterogeneous patient populations [3,4,5,6]. IC-guided approaches have therefore been incorporated into clinical nutrition guidelines and evaluated in interventional studies [3,4,5]. However, IC quantifies energy expenditure, not energy absorption, and does not provide information on the fraction of delivered nutrition that is lost through the gastrointestinal tract [4,5,6].

Gastrointestinal dysfunction, including altered motility, impaired secretion, and feeding intolerance, is common across a wide range of adult patient populations and may compromise nutrient absorption even when nutritional prescriptions are met [1,2]. Empirical data demonstrate that fecal energy losses can be substantial in patients receiving enteral nutrition, indicating that absorption efficiency may be considerably lower than generally assumed [7]. In addition, reference data from adults without overt gastrointestinal disease show marked interindividual variability in fecal energy loss, underscoring that intestinal energy absorption is not uniform even under controlled conditions [8].

Bomb calorimetry of stool samples provides a direct analytical method to quantify fecal energy content and, when combined with detailed intake measurements, enables estimation of intestinal energy absorption [9]. Bomb calorimetry is widely regarded as the reference technique for determining the caloric content of biological samples and has a long-standing role in nutrition and metabolic research [9,10]. Standardized methodological frameworks combining bomb calorimetry with careful monitoring of ingested and excreted calories have been described and allow for robust assessment of nutrient absorption in adult humans [11].

Despite the analytical value of stool bomb calorimetry, its application across adult patient studies appears to be heterogeneous [11]. Differences in study design, patient populations, outcome definitions, and reporting formats may substantially influence measured fecal energy losses and limit comparability between studies [11]. To date, no systematic review has synthesized how bomb calorimetry has been applied to stool samples in adult patient populations, nor critically evaluated the methodological variability that may affect the interpretation of intestinal energy loss and absorption estimates.

This systematic review and meta-analysis aims to evaluate the use of bomb calorimetry for measuring fecal energy loss in adult patient populations, to describe study characteristics and methodological reporting, to quantify intestinal energy absorption and its variability, and to determine whether between-study heterogeneity is explained by the patient populations studied.

## 2. Materials and Methods

### 2.1. Reporting Guidelines

This systematic review was registered with PROSPERO (CRD420261389214) and is reported in accordance with the Preferred Reporting Items for Systematic Reviews and Meta-Analyses (PRISMA) 2020 statement. The search strategy, eligibility criteria and analytical approach were defined in the registered protocol prior to study selection. Two amendments were subsequently registered: a revised and broadened search strategy, and the addition of a post hoc subgroup analysis by patient population. Both are identified as such throughout this report.

### 2.2. Search Strategy

The comprehensive literature search was performed in PubMed, Embase, ClinicalTrials.gov, the Bielefeld Academic Search Engine (BASE; https://www.base-search.net) and the Networked Digital Library of Theses and Dissertations (NDLTD; https://www.ndltd.org). BASE and NDLTD were searched to capture the grey literature and unpublished dissertations. No restriction on publication year was applied. In accordance with the registered protocol, studies published in English, Dutch, Spanish or French were eligible.

For PubMed and Embase, controlled vocabulary terms (MeSH or Emtree) were combined with free-text terms. Boolean operators were used to combine concepts (AND) and synonyms (OR). The PubMed search strategy consisted of: (“Adult”[MeSH] OR adult*) AND (“bomb calorimet*”) AND (“energy absorption” OR “energy digestibility” OR “digestible energy” OR “fecal energy”).

An initial search was performed on 8 September 2025. During peer review, it became apparent that this strategy had limited sensitivity: all search strings required the literal phrase “fecal energy”, “energy absorption”, “energy digestibility” or “digestible energy” and did not include the British spelling “faecal”, nor alternative terms such as “stool energy”, “energy excretion” or “apparent digestibility”; an adult term was also required, which imposed a third restriction. The search was therefore repeated on 28 August 2026 using broadened strings that removed the population restriction, added British orthography and alternative outcome vocabulary, and applied a standard animal-exclusion filter. Complete search strings for both the original and the revised strategy, the date each source was searched, and notes on platform-specific syntax adaptations are provided in Supplementary Table S1. Citation tracking was performed on all included studies and is reported as a separate identification route in the PRISMA flow diagram.

In addition, citation tracking was performed on all included studies to identify additional relevant publications not captured through the database searches.

### 2.3. Eligibility Criteria

Studies were selected based on predefined inclusion and exclusion criteria.

Studies were eligible for inclusion if they:

• Involved human adults (≥18 years).
• Included patients who were hospitalized, in the ICU or post-ICU setting, or free-living.
• Assessed energy absorption, energy digestibility, or fecal energy loss using bomb calorimetry.
• Clearly reported caloric intake, with or without the use of markers or dyes to track food intake.
• Used observational or experimental study designs, including (randomized) controlled trials, randomized) cross-over trials, cohort studies, validation studies, diagnostic accuracy studies, prospective, retrospective, or cross-sectional studies.

Studies were excluded if they:

• Were conducted in animals or in vitro settings.
• Included healthy adults only or children (≤18 years).
• Used calorimetry methods other than stool bomb calorimetry.
• Did not report original results or had no results posted.
• Did not allow a clear assessment of ingested caloric intake.

### 2.4. Study Selection and Risk-of-Bias Assessment

All records identified through the database searches were imported into Rayyan, and duplicates were removed. Title and abstract screening, and subsequent full-text screening, were performed independently by two reviewers (M.A. and E.V.). Registry records and conference abstracts were screened alongside journal articles and excluded where original results were not reported.

**Figure 1:**
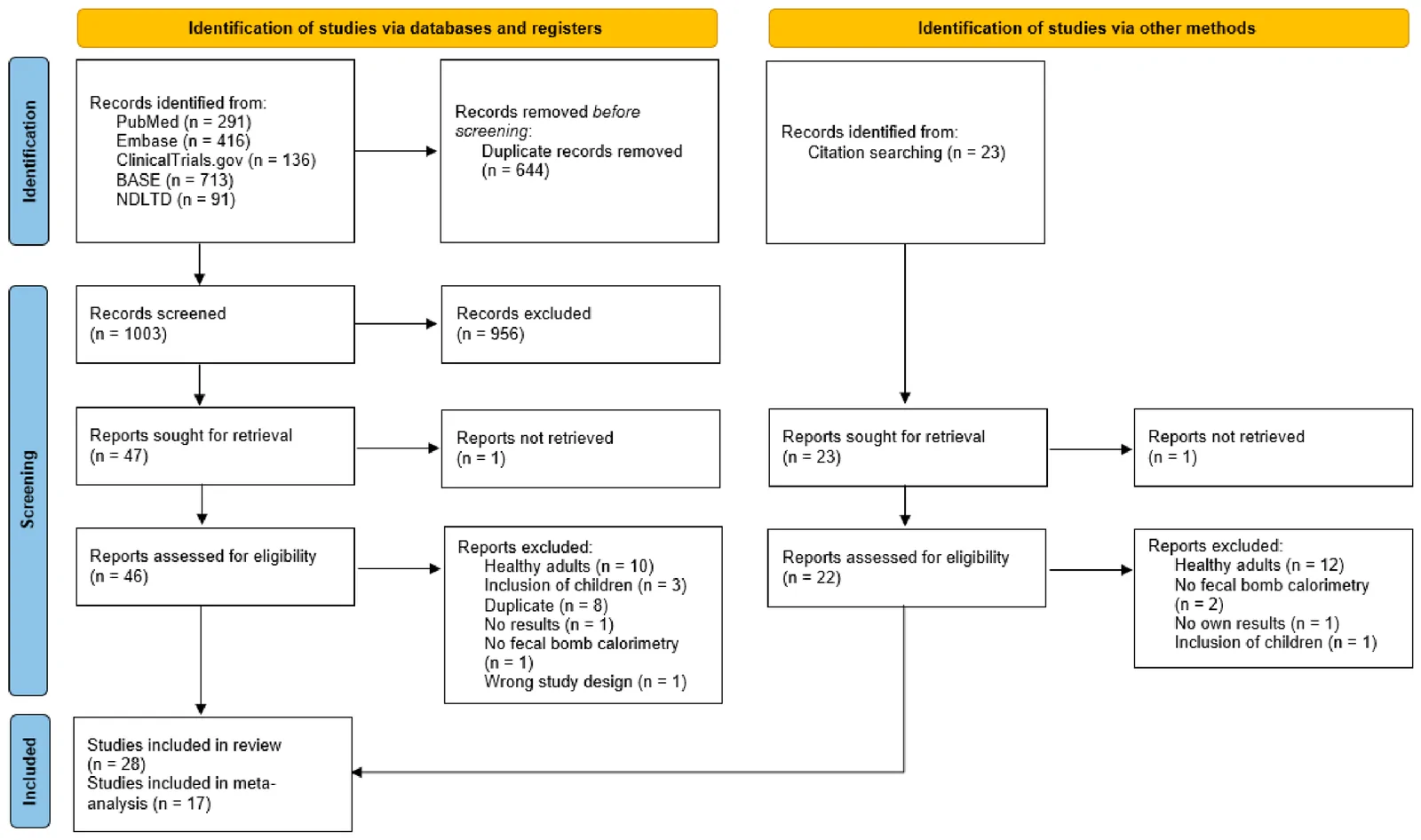
PRISMA flow diagram showing the identification and screening process.

Discrepancies between reviewers were resolved through discussion until consensus was reached. The study selection process and reasons for exclusion are presented in the PRISMA flow diagram (Figure 1).

Because the included studies used different designs, no single risk-of-bias instrument was applicable across the review, and instruments were selected to match the design of the data used in this review rather than the design of the parent study. This distinction matters here: several included studies were interventional, but this review used their baseline or control condition as a descriptive estimate of absorption rather than their intervention effect. Accordingly, the JBI Critical Appraisal Checklist for Analytical Cross-Sectional Studies was applied to studies contributing observational or baseline data, and the JBI Critical Appraisal Checklist for Quasi-Experimental Studies to single-arm before–after studies. One randomized trial was assessed with the Cochrane Risk of Bias 2 tool. One report was a methodological transferability substudy comparing bomb calorimetry between laboratories, a design to which no validated risk-of-bias instrument applies; it was appraised against a set of adapted items describing the completeness of its methodological reporting. This adapted appraisal is not a validated risk-of-bias instrument, does not yield a risk-of-bias judgement, and its item-level ratings are not comparable with those obtained from the JBI checklists or from RoB 2; it is reported separately from the formal appraisals and for transparency only. Each study was appraised against the specific outcome used in this review (intestinal energy absorption or apparent energy digestibility), which is recorded alongside every assessment. Appraisals were performed independently by two reviewers (M.A. and E.V.); agreement was complete and no discrepancies required resolution. Item-level judgements with supporting justifications are provided in Supplementary Table S3. Because JBI checklists are not scored on a common numerical metric, appraisals were used to interpret findings narratively and were not used to weight studies in the meta-analysis.

### 2.5. Data Extraction and Synthesis

Data were extracted using a standardized data extraction form. The following study characteristics were extracted: study source, country, patient population, sample size as enrolled and as analyzed for the absorption outcome, age, sex distribution, comparator, intervention, study design, outcomes and reported results. Where the number of participants contributing to the absorption estimate differed from the number enrolled, the analyzed number was used in the meta-analysis, and the discrepancy was footnoted. Data extraction was performed by one reviewer and verified by a second. In addition, each study was assessed against a structured framework of methodological reporting elements derived from the procedural standards described by Basolo et al. [11]: whether energy intake was quantified concurrently with stool collection, the duration of the collection period, use of a marker of collection completeness, stool handling and storage, homogenization, drying, identification of the calorimeter, calibration, replicate determinations, and clarity of reported units. The completed framework for all included studies is provided as Supplementary Table S2.

Results were synthesized descriptively, focusing on methodological approaches, outcome reporting and sources of heterogeneity.

Meta-analysis: Studies were eligible for quantitative synthesis if they reported, or permitted calculation of, the mean percentage of ingested energy absorbed together with a measure of variance and a sample size. Because the included populations, nutritional regimens and study designs were clinically heterogeneous, a common true effect was not assumed, and a random-effects model was specified. Between-study variance (tau2) was estimated using the Paule–Mandel estimator, and study weights were the inverse of the sum of within-study variance and tau2, with within-study variance derived as SD2/*n*. Where a study reported more than one arm, condition or period, the pre-intervention or control condition was used to avoid double-counting participants. Heterogeneity was quantified using Cochran’s Q, tau2, tau and I2 with a 95% confidence interval, and a 95% prediction interval was calculated to express the range within which the absorption percentage of a future comparable study would be expected to fall. The proportion of between-study variance explained by patient population was quantified as a pseudo-R2, calculated as (tau2 total − tau2 residual)/tau2 total, where tau2 total is the between-study variance estimated in the model without moderators and tau2 residual is that estimated in a mixed-effects model including patient population as a moderator, both obtained with the same estimator. The reduction in Cochran’s Q was not used to quantify explained heterogeneity. Funnel plot asymmetry was assessed using Egger’s regression test, with the interpretive caveat that such tests have low power and are unreliable in the presence of substantial heterogeneity. Sensitivity analyses comprised leave-one-out estimation, influence diagnostics, and exclusion of studies contributing pooled subgroup data.

Subgroup analysis: Following inspection of the pooled result, a post hoc subgroup analysis was performed comparing studies of short bowel syndrome and intestinal failure with all other patient populations, and a test for subgroup differences was calculated. This analysis was not prespecified and is reported as exploratory. Analyses were performed in R version 4.4.2 (R Foundation for Statistical Computing, Vienna, Austria) using the metafor package version 5.0-1, with readxl 1.5.0, readr 2.1.5, dplyr 1.2.1, ggplot2 4.0.3 and writexl 2.0.1 for data handling and graphics.

## 3. Results

### 3.1. Study Selection

The revised literature search identified 1647 records through database searching: PubMed (*n* = 291), Embase (*n* = 416), ClinicalTrials.gov (*n* = 136), BASE (*n* = 713) and NDLTD (*n* = 91). After removal of 644 duplicate records, 1003 records remained for title and abstract screening.

Of these, 956 were excluded at title and abstract screening, and 47 reports were sought for retrieval. One report could not be obtained. The remaining 46 reports were assessed for eligibility, of which 24 were excluded: healthy adults only (*n* = 10), inclusion of children (*n* = 3), duplicate publication (*n* = 8), no results reported (*n* = 1), no fecal bomb calorimetry (*n* = 1), and ineligible study design (*n* = 1). Twenty-two studies were included from database searching [7,8,12,13,14,15,16,17,18,19,20,21,22,23,24,25,26,27,28,29,30,31].

In addition, 23 records were identified through citation tracking, of which 22 were retrieved and assessed in full. Reports that could not be retrieved are listed in Supplementary Table S4. Sixteen were excluded because they studied healthy adults only (*n* = 12), did not use fecal bomb calorimetry (*n* = 2), reported no original results (*n* = 1), or included children (*n* = 1), leaving six included studies [32,33,34,35,36,37].

In total, 28 studies met all inclusion criteria and were included in the systematic review. Seventeen of these reported or permitted calculation of percentage energy absorption together with a measure of variance and were included in the meta-analysis [7,8,12,16,20,22,23,24,25,28,31,32,33,34,35,36,37]. The study selection process and reasons for exclusion are summarized in the PRISMA flow diagram (Figure 1).

### 3.2. Study Characteristics

The characteristics of the 28 included studies are summarized in Table 1. The studies were published between 1981 and 2026 and comprised a heterogeneous set of designs, including cross-sectional and prospective observational studies, single-arm and non-randomized interventional studies, randomized and cross-over trials, and diagnostic accuracy studies. Sample sizes ranged from 6 to 89 participants. All studies were conducted in high-income settings, which limits generalizability to other healthcare contexts.

The clinical conditions represented were short bowel syndrome and intestinal failure, chronic pancreatitis and pancreatic cancer, celiac and refractory celiac disease, Crohn’s disease, critical illness requiring enteral nutrition, severe obesity, HIV infection, and mixed malabsorptive and non-malabsorptive disease. Studies of healthy adults alone were excluded; healthy participants appear only as comparator groups within otherwise eligible studies.

### 3.3. Fecal Energy Losses and Energy Absorption

All included studies reported quantitative measurements of fecal energy content obtained through bomb calorimetry. Across the 17 studies contributing to the meta-analysis, mean absorption ranged from 50.0% in patients with short bowel syndrome and intestinal failure to 91.9% in prediabetic patients with obesity.

Absorption estimates clustered by clinical condition rather than being distributed continuously. Studies of short bowel syndrome and intestinal failure reported estimates between 50.0% and 69.0%, whereas studies of critical illness, celiac disease, Crohn’s disease and metabolic conditions reported estimates between 82.2% and 91.9%. Studies of pancreatic disease fell between these groups, at 72.9% and 73.9%. This pattern is examined formally in Section 3.7.

### 3.4. Outcome Reporting Heterogeneity

Marked heterogeneity was observed in outcome definitions and reporting. Outcomes derived from stool bomb calorimetry were reported as fecal energy content, absolute fecal energy loss over a defined period, percentage of ingested energy excreted, or derived estimates of intestinal energy absorption, and units varied between studies. Seventeen of 28 studies reported or permitted calculation of percentage energy absorption together with a measure of variance. Reporting of the technical procedures underlying these measurements was also incomplete (Supplementary Table S2). Energy intake was quantified concurrently with stool collection in 25 of 28 studies, and the make and model of the calorimeter were identified in 20 of 28. A marker or procedure to establish completeness of stool collection was described in only four of 28 studies. Calibration procedures and replicate determinations were each reported in one of 28. Whether fecal energy was expressed per unit wet or dry weight was frequently not stated.

### 3.5. Meta-Analysis

**Figure 2:**
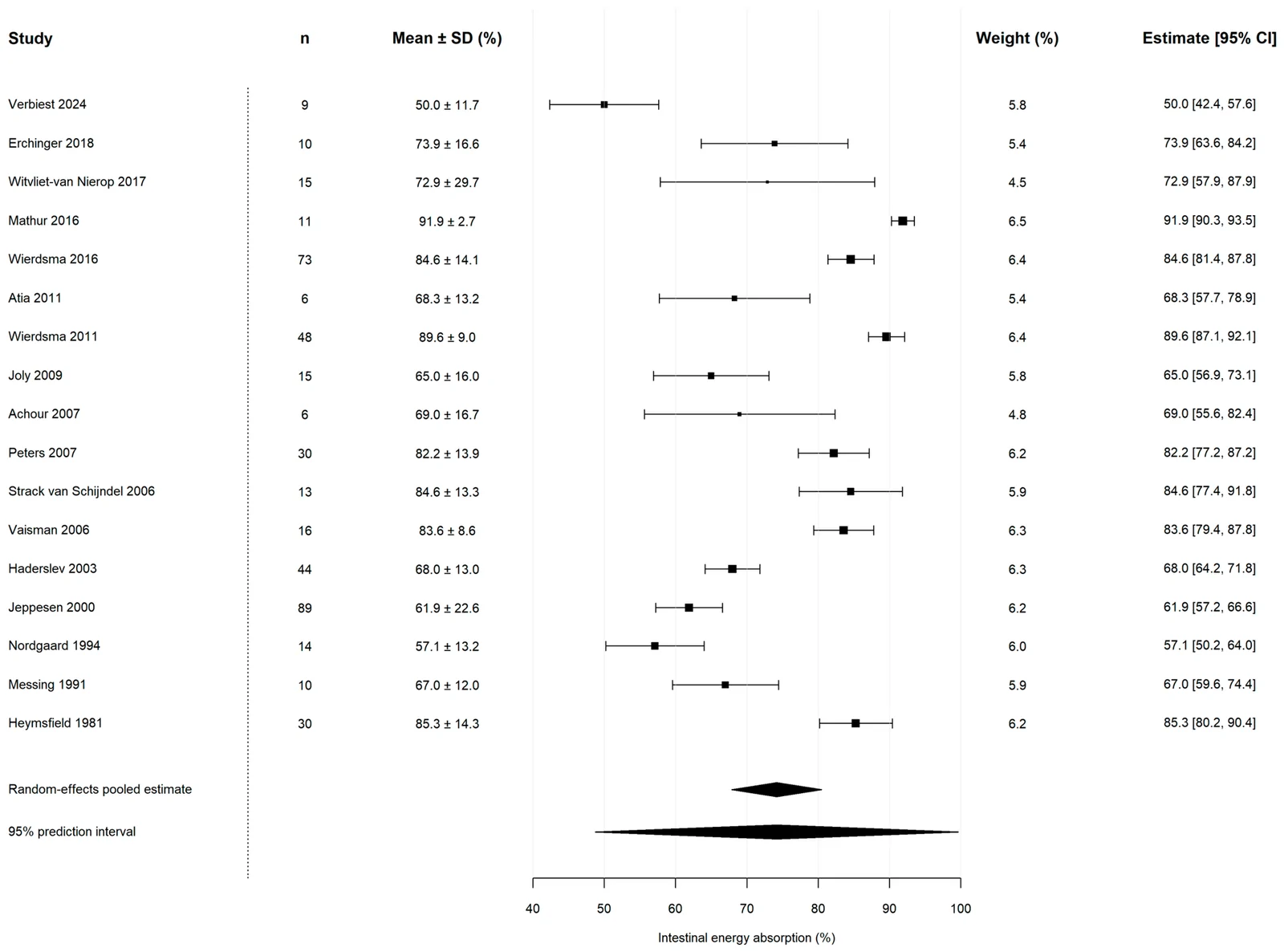
Forest plot of intestinal energy absorption in the 17 studies contributing to the meta-analysis (439 participants) [7,8,12,16,20,22,23,24,25,28,31,32,33,34,35,36,37]. Squares represent study estimates, sized by weight; horizontal lines are 95% confidence intervals. The lower diamond shows the random-effects pooled estimate and the widest diamond the 95% prediction interval.

Seventeen of the 28 studies were eligible for quantitative synthesis, contributing 439 participants. Studies were excluded from the meta-analysis because percentage energy absorption was not reported and could not be calculated, or because no measure of variance was reported or derivable. The random-effects pooled estimate of intestinal energy absorption was 74.2% (95% CI 67.9–80.5). Between-study heterogeneity was substantial: Q = 459.8 (df = 16, *p* < 0.001), tau2 = 135.2, tau = 11.6 percentage points, and I2 = 96.3% (95% CI 93.3–98.4). The 95% prediction interval was 48.8% to 99.6%. Individual study estimates and weights are shown in Figure 2.

The magnitude of I2 indicates that almost all observed variation between studies exceeds what would be expected from within-study sampling error alone. Considered without reference to the populations studied, the pooled estimate therefore describes the centre of a widely dispersed distribution rather than a value applicable to any particular patient group. Sensitivity analyses supported the stability of the pooled estimate. Leave-one-out estimation yielded values between 73.0% and 75.8%, and no single study was influential on formal diagnostics. Restricting the analysis to the ten studies whose estimates did not require pooling of reported subgroups gave 70.1% (95% CI 61.1–79.2). Egger’s regression test did not indicate significant funnel plot asymmetry (t = −2.00, df = 15, *p* = 0.064); this test has low power and is unreliable at this level of heterogeneity, and any asymmetry is in any case confounded by study population, since the populations with the lowest absorption were also those studied in the smallest samples.

### 3.6. Risk-of-Bias Assessment

Risk of bias was assessed using instruments selected to match the design of the data used in this review. A summary of item-level judgements across all studies is shown in Supplementary Figure S1. The JBI Critical Appraisal Checklist for Analytical Cross-Sectional Studies was applied to 21 studies and the JBI Checklist for Quasi-Experimental Studies to five single-arm before–after studies; one randomized trial was assessed with RoB 2. One methodological transferability substudy was appraised against adapted methodological items, since no validated risk-of-bias instrument applies to that design; these ratings describe completeness of reporting rather than risk of bias, are not comparable with the JBI or RoB 2 judgements, and are therefore presented separately in Supplementary Figure S1 and Supplementary Table S3. Item-level judgements, supporting justifications and the specific review outcome assessed for each study are provided in Supplementary Table S3. Across studies, items relating to measurement of the outcome and to statistical analysis were generally well satisfied, reflecting the analytical robustness of bomb calorimetry itself. The recurring weaknesses were in the reporting of eligibility criteria, in identification of and adjustment for confounding, and in the description of exposure measurement. These limitations concern the completeness of reporting more than the conduct of the measurements, and they bear directly on the interpretation of the pooled estimate.

### 3.7. Subgroup Analysis by Patient Population (Post Hoc)

Because heterogeneity remained substantial and the distribution of study estimates appeared clustered by clinical condition, a post hoc subgroup analysis was performed comparing studies of short bowel syndrome and intestinal failure with all other patient populations. This analysis was not prespecified in the registered protocol and is reported as exploratory.

Eight studies enrolled patients with short bowel syndrome or intestinal failure. Their pooled absorption was 62.9% (95% CI 57.4–68.5), with moderate residual heterogeneity (I2 = 70.1%, Q = 23.4, df = 7, *p* = 0.001); individual estimates ranged from 50.0% to 69.0%. The remaining nine studies pooled to 85.1% (95% CI 81.2–88.9), with substantial residual heterogeneity (I2 = 83.8%).

**Figure 3:**
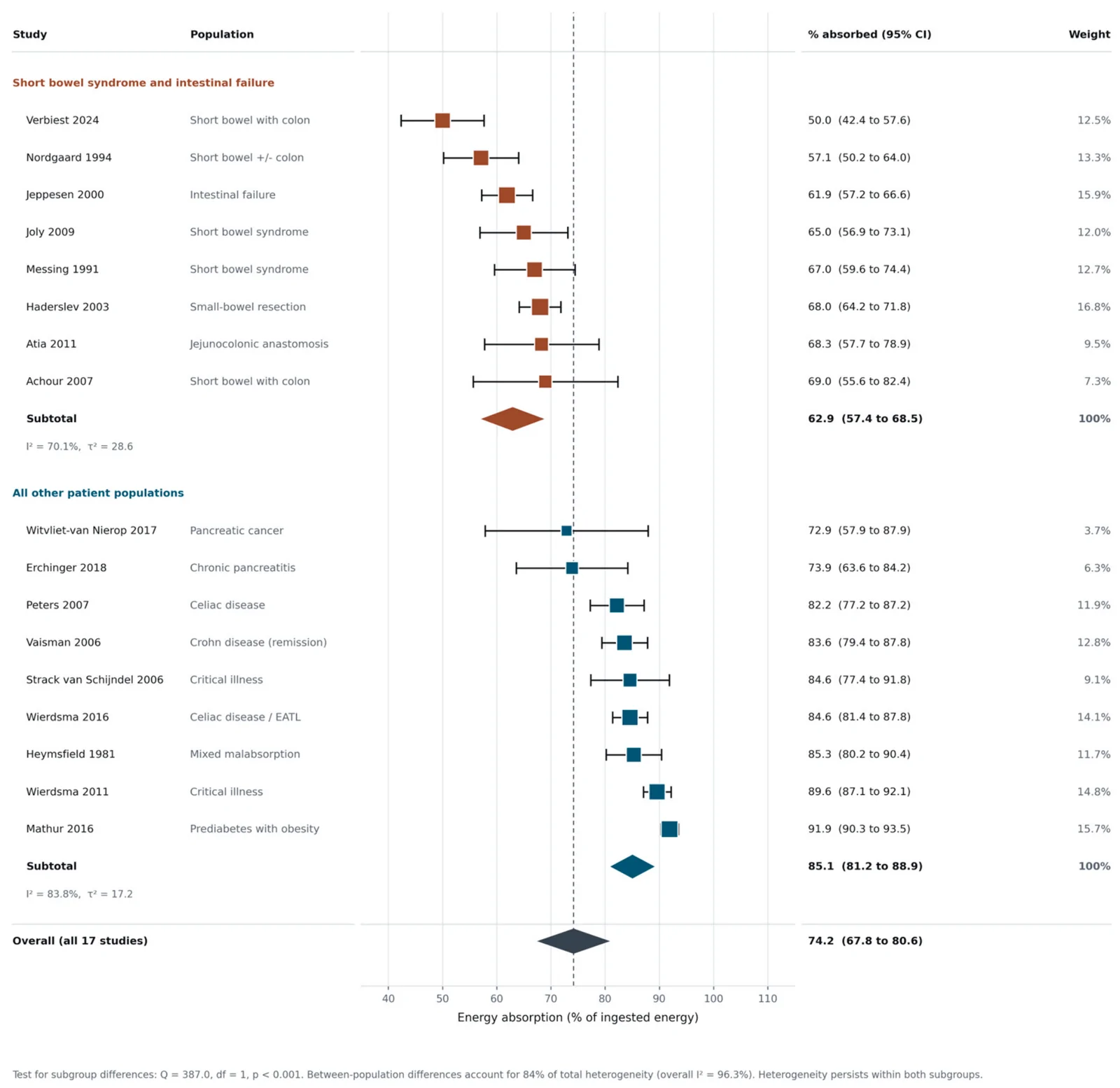
Forest plot of the post hoc subgroup analysis by patient population, comparing short bowel syndrome and intestinal failure with all other patient populations [7,8,12,16,20,22,23,24,25,28,31,32,33,34,35,36,37]. Diamonds represent subgroup and overall random-effects estimates with 95% confidence intervals.

The difference between the two subgroups was 22.2 percentage points, and the test for subgroup differences was significant (Q = 387.0, df = 1, *p* < 0.001). Including patient population as a moderator reduced the between-study variance from tau2 = 135.2 to tau2 = 24.0, a pseudo-R2 of 82%. Population therefore explains most, but not all, of the variability in reported absorption; heterogeneity persists within both subgroups and is not accounted for by this classification. With 17 studies distributed across two subgroups, this proportion is itself estimated imprecisely and should be read as indicating that population is the dominant source of heterogeneity rather than as an exact quantity. Subgroup estimates are shown in Figure 3.

Residual variation within the short bowel and intestinal failure subgroup is itself interpretable. The lowest estimates came from cohorts with the most extensive intestinal loss or the greatest dependence on parenteral support, and the highest from cohorts with a preserved colon in continuity. Remnant anatomy rather than diagnosis alone is therefore likely to determine absorption within this group, although the number of studies is too small to test this formally.

A within-study comparison supports the between-population contrast. Peters et al. studied 15 patients with celiac disease, nine with refractory celiac disease and six with short bowel syndrome using a single protocol, a single calorimeter and a single laboratory. Absorption was 89.2% in the celiac group and 82.3% in the refractory celiac group (86.6% combined), against 64.3% in the short bowel group, a difference of 22.3 percentage points that closely matches the between-subgroup difference observed across the review. Because equipment, intake ascertainment, laboratory and investigators were identical across these groups, this comparison is not susceptible to the between-study methodological differences that might otherwise explain the subgroup effect.

## 4. Discussion

This systematic review and meta-analysis synthesized 28 studies published between 1981 and 2026 evaluating the use of bomb calorimetry for measuring fecal energy loss and absorption in adult patient populations. The principal finding is that intestinal energy absorption varies widely between adult patients, and that most of this variation is attributable to the population studied rather than being unexplained.

Pooled across the 17 studies contributing quantitative data, absorption was 74.2% (95% CI 67.9–80.5) with an I2 of 96.3% and a prediction interval spanning 48.8% to 99.6%. Taken alone, that result supports only a negative conclusion: no single value describes intestinal energy absorption in adult patients. The post hoc subgroup analysis is more informative. Patients with short bowel syndrome or intestinal failure absorbed 62.9% of ingested energy against 85.1% in all other populations, and this single distinction accounted for approximately 82% of the between-study variance that had otherwise appeared unexplained.

The magnitude of this difference has direct clinical meaning. A patient with intestinal failure prescribed 2000 kcal absorbs approximately 1260, whereas a patient with celiac disease or critical illness receiving the same prescription absorbs approximately 1700. A deficit of this size is invisible to any monitoring approach based on delivered energy alone. The within-study comparison in Peters et al., where celiac and short bowel patients studied under an identical protocol differed by 22.3 percentage points, indicates that this reflects physiology rather than differences between research groups.

Heterogeneity nonetheless persists within both subgroups (I2 = 70.1% and 83.8%), so population is not a complete explanation, and three further sources of variation are identifiable from the included studies. First, cohorts were assembled differently: Haderslev et al. enrolled only patients with malabsorption exceeding 2000 kJ/d, so that estimate is conditioned on a criterion that guarantees impaired absorption, whereas cohorts of critically ill patients receiving enteral nutrition had no such entry criterion. Second, the denominator differed: studies that quantified intake by bomb calorimetry of a duplicate diet measured ingested energy directly, whereas those relying on food diaries or the declared composition of enteral formulae estimated it, and error in the denominator propagates directly into the absorption percentage without appearing in the reported variance. Third, whether fecal energy was expressed per unit wet or dry weight was frequently unstated, and fecal water content varies widely in precisely these populations.

The structured assessment of methodological reporting (Supplementary Table S2) quantifies how incompletely these procedures are described. Calibration of the calorimeter and replicate determinations were each reported in one of 28 studies, and a marker of stool collection completeness in four of 28. It is therefore not possible to determine from the published literature whether technical procedures are standardized across laboratories. This is itself a finding: the degree of technical standardization in this field is currently unverifiable from the published record, which is a stronger argument for reporting standards than any assumption of uniformity would be.

Risk-of-bias appraisal identified recurring weaknesses in the specification of eligibility criteria, in the identification of confounding, and in the description of how intake was measured, rather than in the calorimetric measurement itself. These are limitations of reporting more than of conduct, but they constrain what can be concluded from the pooled data and reinforce the case for a minimum reporting standard.

These findings have specific implications for clinical practice. The included studies suggest four contexts in which measured rather than assumed absorption could alter clinical reasoning. First, in intestinal failure, absorption measured by bomb calorimetry has already been used to define the condition itself, and comparable measurements serve as endpoints in trials of glucagon-like peptide-2 analogues, where the question is whether parenteral support can be reduced; the subgroup estimate of 62.9% provides an expected value for this population, although the residual heterogeneity within it means the figure should be treated as a central tendency rather than a value applicable to an individual. Second, in critical illness, energy delivery is titrated against measured or predicted expenditure with no accounting for absorption; the higher pooled estimate in this group suggests the resulting error is smaller than in intestinal failure, but it is not zero. Third, in exocrine pancreatic insufficiency, fecal energy loss offers an objective endpoint against which enzyme replacement can be titrated. Fourth, several included studies used bomb calorimetry as the reference standard against which simpler bedside surrogates were evaluated, such as fecal weight and fasting plasma citrulline, and this reference role is where the technique is most immediately applicable, since it requires complete stool collection and is not a bedside test.

These are potential applications rather than demonstrated ones. No included study evaluated whether measuring absorption and acting on the result changes nutritional status, function or any patient-centred outcome. Such a trial has not been performed, and it is the necessary next step before fecal energy assessment can be recommended in routine care.

From a methodological perspective, the reporting deficits identified here are addressable. Building on the procedural framework described by Basolo et al. [11], we propose that studies reporting stool bomb calorimetry in adult patients report the following as a minimum. The primary outcome should be the percentage of ingested energy absorbed, reported as mean with standard deviation and sample size, alongside rather than instead of absolute fecal energy loss, since only the percentage is comparable across populations with differing intakes. The method of intake ascertainment should be stated explicitly, as bomb calorimetry of a duplicate diet, weighed food records, and the declared composition of enteral formulae are not interchangeable in accuracy. Studies should report the duration of the balance period, the use and type of any collection marker, the criteria used to judge collection completeness, and the number of participants contributing complete collections with reasons for any exclusion. Sample handling should specify storage temperature, homogenization, drying method and whether energy is expressed per unit wet or dry weight. The calorimeter, its calibration standard and the number of replicate determinations should be identified. Finally, in short bowel syndrome and intestinal failure, remnant small bowel length and the presence of colon in continuity should be reported, since these appear to determine absorption within that population. Reporting per-participant data, or at minimum full variance estimates, would allow future syntheses to include studies that must currently be excluded: in this review, 11 of 28 studies could not be pooled for precisely this reason.

###  Limitations and Gaps

This systematic review has several limitations. First, the original search strategy had limited sensitivity, as described in Section 2.2; although it was subsequently broadened and re-run, and the revised strategy retrieved all previously included studies, the possibility that further eligible studies remain unidentified cannot be excluded. Two reports could not be obtained because no full text was available; one of these (Woolf et al., Dig Dis Sci 1987 [38]) reports intestinal energy absorption measured by bomb calorimetry in adults with short bowel syndrome and would appear to have met the eligibility criteria. Second, the subgroup analysis was performed post hoc after inspection of the pooled result; although the difference between populations is large and supported by a within-study comparison, it requires confirmation in prospectively defined analyses. Third, heterogeneity remained substantial within both subgroups, so patient population does not fully explain the observed variation, and subgroups other than short bowel syndrome and intestinal failure contained too few studies to support separate pooled estimates. Fourth, eleven of the 28 included studies could not contribute to the quantitative synthesis because percentage absorption or a measure of variance was not reported and could not be derived, so the pooled estimate rests on a subset of the identified evidence. Fifth, several included studies reported methodological details incompletely, limiting evaluation of technical heterogeneity; one included report, a methodological transferability substudy, could be appraised only against adapted items rather than a validated instrument, so its appraisal is not comparable with the others. Sixth, all included studies were conducted in high-income settings. Finally, the included studies span more than four decades, during which calorimetric technology and nutritional practice have both evolved.

## 5. Conclusions

Bomb calorimetry provides a direct and analytically robust method for quantifying fecal energy content and estimating intestinal energy absorption in adult patients. Pooled across 17 studies, absorption was 74.2% (95% CI 67.9–80.5) with substantial heterogeneity (I2 = 96.3%) and a prediction interval spanning 48.8% to 99.6%. This variability is largely structured rather than random: patient population accounted for approximately 82% of the between-study variance. Patients with short bowel syndrome or intestinal failure absorbed 62.9% (95% CI 57.4–68.5) of ingested energy, more than 20 percentage points less than other adult patient populations, which absorbed 85.1% (95% CI 81.2–88.9).

Prescribed energy intake should therefore not be assumed to equal absorbed energy, particularly in patients with short bowel syndrome or intestinal failure, in whom approximately one third of ingested energy is lost. Heterogeneity nonetheless persists within populations, so these values describe groups rather than individuals, and measurement remains preferable to assumption where the result would change management. The principal barrier to further synthesis in this field is descriptive rather than analytical: outcome definitions, units and variance reporting differ to a degree that excluded 11 of 28 otherwise eligible studies from quantitative synthesis, and technical procedures are reported too incompletely to establish whether they are standardized. Adoption of a minimum reporting set, prospective studies applying it consistently, and trials evaluating whether acting on measured absorption improves patient outcomes are the necessary next steps before fecal energy assessment can be recommended for routine clinical use.

## Figures and Tables

**Table 1:** Characteristics of included studies. Characteristics of the 28 studies included in the systematic review, listed alphabetically by first author. Stool bomb calorimetry was performed in all included studies and is therefore not repeated in the Intervention column, which lists only additional interventions. “Reported results” gives the outcomes as published; “Result used in this review” gives the value extracted for synthesis, with the derivation shown where the two differ. NA = not applicable; NR = not reported.

Study Source	Country	Patient Population	Total *n*	*n* with Data	Age	% Male	Comparator	Intervention	Study Design	Study Outcomes	Reported Results	Result Used in This Review	Derivation
Achour, 2007 [32]	France	Patients with SBS	6	6	60.5 ± 17.6	66.7%	Diet quantified according to French Food Composition Tables	Analysis of bacterial stool fractions	Observational comparative metabolic balance study	Fecal energy loss (kJ/day) & apparent energy digestibility (%)	FE: 3960 ± 2396; EA: 69.0 ± 16.7%	69.0 ± 16.7%	SD calculated from SEM
Andreae, 2026 [30]	USA	Male patients with HIV	19	19	48.5 ± 7.9	100.0%	1-month food frequency questionnaire (FFQ) analyzed by NutritionQuest (habitual energy intake)	NA	Cross-sectional observational comparative study	Energy intake, estimated (kcal) & fecal energy (kcal)	Intake: 2418.33 ± 1095.60; FE: 5457.18 ± 415.43	Not derivable	Unit unclear; cannot be kcals and kcal
Atia, 2011 [31]	USA	Patients with SBS	6	6	48.3 ± 15.6	50.0%	Dietary constituent analysis of duplicate of standardized meal	Pectin supplementation	Single-arm trial	Energy absorption (kcal/day & %)	1768 ± 555 = 68.3 ± 13.2%	68.3 ± 13.2%	–
Brownell, 2020 [13]	USA	Patients with chronic pancreatitis	24	24	47.1 ± 10.4	43.5%	Three-day average diet intake analysis (Nutrient Data System)	PERT	Open-label cohort study	Fecal energy (cal/g)	Mean kcal consumed/day: 1714 ± 641 kcal/day; mean cal excreted/g: 5728 ± 717 cal/g; mean g of stool/day: 372 ± 146 g/day -> mean kcal excreted per day: 2131 kcal/day	Not derivable	–
Eliasson, 2022 [15]	Denmark	Patients with SBS	8	8	61 ± 13.8	37.5%	Bomb calorimetry of duplicate dietary intake	Apraglutide	Single-arm trial	Absolute change in intestinal energy absorption (kJ/day)	mean (95% CI) = 1095 (196–1994)	Not derivable	–
Erchinger, 2018 [35]	Norway	Patients with chronic pancreatitis, diagnosed according to the Layer score	10	10	66.7 ± 17.78	31.8%	Energy content of diet calculated via validated tables	NA	Non-randomized controlled trial	Fecal energy loss (kcal/day) & coefficient of energy absorption (%)	Control: FE = 201.3 ± 73.9; CEA = 90.0 ± 3.1; CP: FE = 471.0 ± 342.2; CEA = 73.9 ± 16.6	73.9 ± 16.6%	–
Haderslev, 2003 [16]	Denmark	Patients who have undergone small-intestinal resection	44	44	50.6 ± 12.3	45.5%	Bomb calorimetry of duplicate dietary intake	NA	Cross-sectional observational study	Absorbed energy (kJ/day) grouped by intestinal net energy absorption (NEA) relative to the BMR and fecal energy (kJ/day) grouped by intestinal NEA relative to BMR	Mean: dietary intake: 12381 kJ/day; fecal output: 4358 kJ/day; energy absorption: 8022 kJ/day; weighted mean: dietary intake: 12200 ± 4230 kJ/day; fecal output: 4126 ± 3158; energy absorption: 8078 ± 2057	68 ± 13%	Weighted mean calculated and estimated SD
Heydorn, 1999 [17]	Denmark	Patients with SBS	4	4	63.25 ± 14.75	50.0%	Bomb calorimetry of duplicate dietary intake	Cholylsarcosine (bile acid replacement therapy)	Randomized cross-over trial	Energy absorption at baseline in percentage of diet and in MJ/day	11.0 MJ/day; energy absorption = 66%	66%	–
Heymsfield, 1981 [37]	USA	Patients with malabsorption and non-malabsorption diseases	30	30	NR	NR	Constant diets weighed and prepared in a metabolic kitchen	NA	Cross-sectional observational study	Fecal energy loss (kcal/day) & energy digestibility (%)	Malabsorption: FE = 493 ± 275; AE = 73 ± 12; non-malabsorption: FE = 74 ± 48; AE = 96 ± 3	85.3 ± 14.3%	Weighted mean energy absorption and SD calculated
Jeppesen, 1998 [19]	Denmark	Patients with SBS with or without a preserved colon	19	19	51 ± 16.2	47.4%	Bomb calorimetry of duplicate dietary intake	2 diets: LCT & LCT + MCT	Randomized cross-over trial	Absorbed energy (MJ/day) on different high-fat diets (LCT & LCT + MCT) & fecal energy (MJ/day) on different high-fat diets	No colon, LCT: AE = 4819 ± 1912; FE = 5983 ± 3122; no colon, LCT + MCT: AE = 5212 ± 1556; FE = 6094 ± 3246; colon, LCT: AE = 4496 ± 1298; FE = 5139 ± 0969; colon, LCT + MCT: AE = 5805 ± 1566; FE = 4220 ± 1286	50.1%	Mean energy absorption calculated
Jeppesen, 2000 [36]	Denmark	Patients with intestinal insufficiency and intestinal failure with or without home parenteral nutrition	89	89	49 ± 11.5	38.2%	Bomb calorimetry of duplicate dietary intake	NA	Cross-sectional observational study	Intestinal energy absorption (MJ/day) & fecal energy excretion (MJ/day)	Non-HPN: Intestinal EA = 71% (58–78); FE = 3.20 (2.26–4.73); HPN: Intestinal EA = 49% (40–76); FE = 4.16 (1.28–5.61)	61.9 ± 22.6%	Estimated mean and SD from median and IQR
Joly, 2009 [23]	France	Patients with SBS	15	15	53.6 ± 12.9	40.0%	3-day measurement and calculation of diet with Bilnut software, Version 4.0 (Nutrisoft, Cérelles, France)	Tube feeding	Randomized cross-over trial	Energy absorption (net) (kcal/day) & energy absorption (%)	EA = 1638 ± 458 = 65 ± 16%	65 ± 16%	–
Mathur, 2016 [12]	USA	Prediabetic patients with obesity	11	11	47 ± 9	18.2%	Bomb calorimetry of customized daily meals	NA	Single-arm trial	Percentage of kcal in stool vs. ingested to calculate the average number of calories absorbed daily by each participant	Total population: 8.1 ± 2.7 to 8.3 ± 3.8	91.9 ± 2.7%	–
Messing, 1991 [25]	France	Patients with SBS	10	10	47 ± 15	80.0%	Bomb calorimetry of duplicate dietary intake and first meal with red carmine	NA	Cross-sectional observational study	Net digestive energy absorption (%)	67 ± 12%	67 ± 12%	–
Naimi, 2019 [18]	Denmark	Patients with SBS	16	10	62.4 ± 7.5	50.0%	Bomb calorimetry of duplicate dietary intake	Glepaglutide	Randomized cross-over trial	Change from baseline in absolute energy absorption (kJ/day)	−377 (95% CI = −1234–481)	Mean energy absorption in change from baseline = −377 ± 1199 kJ/day	Estimated SD from 95% CI
Nielsen, 1995 [29]	Denmark	Patients with liver cirrhosis	15	5	50 ± 11.6	66.7%	Bomb calorimetry of duplicate dietary intake	Refeeding	Single-arm trial	Fecal energy loss during refeeding per kg bodyweight (kJ/kg/day)	11 ± 3 (SE)	Fecal energy loss: 11 ± 6.7 kJ/kg/day	Estimated SD from SE
Nordgaard, 1994 [33]	Denmark	Patients with SBS	14	14	61.1 ± 10.4	42.9%	Standardized 40:40 carb: fat diet, based on habitual intake	High-fat vs. high-carb diet	Randomized cross-over trial	Energy absorbed in standardized 40:40 diet	With colon: 61 ± 4%; without colon: 52 ± 6%	57.1 ± 13.2%	Estimated SD from SE and weighted mean + SD from both groups
Peters, 2007 [20]	the Netherlands	Patients with recently diagnosed celiac disease, refractory celiac disease, or SBS	30	30	51.09 ± 14.76	33.3%	Single fasting plasma citrulline concentration	NA	DTA study	Intestinal energy absorption capacity (%)	Control: 90.3 ± 3.5; CD: 89.2 ± 3.4; RCD: 82.3 ± 11.7; SBS: 64.3 ± 18.2	82.2 ± 13.9%	Weighted mean energy absorption and SD calculated
Pinar, 2025 [14]	Denmark	Patients with SBS	10	10	54.6 ± 15.9	50.0%	Bomb calorimetry of duplicate dietary intake	Glepaglutide	Single-arm trial	Absolute change in intestinal energy absorption (kJ/day) & improvement from baseline in percentage	1038 (192–1883); improvement of 23%	Energy absorption in absolute change from baseline, not comparable	–
Qian, 2024 [21]	USA	Patients with severe obesity who will undergo MBS in the near future	67	67	41.94 ± 9.28	11.9%	Dietary intake questionnaire (ASA24 dietary recall)	NA	Prospective cohort study	Relative fecal energy content for number of months past surgery	No exact numbers (figures, but tables are not reported)	Not derivable	–
Strack van Schijndel, 2006 [7]	the Netherlands	Stable adult ICU patients, dependent on enteral nutrition and on mechanical ventilation	13	13	61.8 ± 17.1	53.8%	Enteral nutrition (three types with known caloric content)	NA	Prospective observational study	Mean fecal energy (kcal/day) & mean total energy absorption (%)	FE = 301 ± 259; AE = 84.6 ± 13.3	84.6 ± 13.3%	–
Vaisman, 2006 [24]	Israel	Patients with Crohn’s disease in remission	16	16	33.2 ± 10.7	62.5%	3-day food diary with activated charcoal on first and fourth day	NA	Cross-sectional observational study	Energy malabsorption (%) & energy absorption (%)	EM = 16.4 ± 8.6%; EA = 83.6 ± 8.6%	83.6 ± 8.6%	EA calculated from energy malabsorption
Verbiest, 2024 [22]	Belgium, Denmark	Patients with SBS	9	9	46.8 ± 17.4	22.2%	Bomb calorimetry of duplicate dietary intake	Apraglutide	Single-arm open-label phase 2 trial	Relative energy absorption (%) with 95% CI	50 (41–59)	50.0 ± 11.7%	Estimated SD from 95% CI
Verbiest, 2025 [27]	Belgium, Denmark	Patients with SBS	7	7	46.8 ± 14.5	14.3%	Bomb calorimetry of duplicate dietary intake	NA	Methodological transferability substudy, nested within a single-arm open-label phase 2 trial	Transferability and agreement of stool bomb calorimetry (fecal output, J/day)	Copenhagen: 4483 ± 1748; Leuven: 4504 ± 1739; ICC: 0.997	Not derivable	–
Wierdsma, 2011 [8]	the Netherlands	Stable adult ICU patients, dependent on enteral nutrition and on mechanical ventilation	48	48	63.4 ± 15.4	58.3%	No residue enteral nutrition	NA	DTA study	Fecal energy loss (kcal/day) & energy absorption capacity (%)	Normal stools: FE = 146.4 ± 86.7; AE = 93.1 ± 4.1; diarrhea: FE = 445.5 ± 201.3; AE = 76.5 ± 10.6	89.6 ± 9.0%	Weighted mean energy absorption and SD calculated
Wierdsma, 2016 [28]	the Netherlands	Patients with (complicated) celiac disease and EATL	73	73	54.9 ± 14.3	44.6%	4-day food diary calculated with a food calculation program	NA	Cross-sectional observational study	Fecal energy (kcal/day) & energy absorption (%)	FE: 404.1 ± 417.0; EA: 84.6 ± 14.1%	84.6 ± 14.1%	Mean age and percentage male for the whole group (n = 92; some feces samples were not available) & weighted mean energy absorption and SD calculated
Witvliet-van Nierop, 2017 [34]	the Netherlands	Patients with locally advanced pancreatic cancer	16	15	60.3 ± 9.5	50.0%	Four-day food diary of intake with calculations of a computerized food calculation program	NA	Cross-sectional observational study	Fecal energy loss (kcal/day) & median of energy absorption capacity (%)	FE = 324 (198–597); EA = 82.9 (47.8–87.9)	72.9 ± 29.7%	Mean and SD from median and IQR estimated
Woolf, 1983 [26]	Canada	Patients with SBS	8	8	46 ± 12.4	25.0%	Bomb calorimetry of duplicate dietary intake	High-fat vs. high-carb diet	Randomized cross-over trial	Mean energy absorption	0.65	65%	–

Abbreviations: AE, absorbed energy; CEA, coefficient of energy absorption; EA, energy absorption; EATL, enteropathy-associated T-cell lymphoma; FE, fecal energy; HPN, home parenteral nutrition; LCT, long-chain triglycerides; MCT, medium-chain triglycerides; NA, not applicable; NR, not reported; PERT, pancreatic enzyme replacement therapy; SBS, short bowel syndrome. Where the number of patients with data differs from the total sample size, the former was used in the meta-analysis: Naimi, 2019 [18] (10 of 16); Nielsen, 1995 [29] (5 of 15); Witvliet-van Nierop, 2017 [34] (15 of 16).

## Data Availability

All data extracted and analyzed in this review are derived from the published studies cited and are provided in Supplementary Tables S1–S4. The analysis code is provided per request.

## Supplementary Materials

The following supporting information can be downloaded at: https://www.mdpi.com/article/10.3390/nu18182976/s1. Table S1: Search strategies for the original and revised searches, with the date each source was searched and notes on platform-specific syntax; Table S2: Methodological reporting of stool bomb calorimetry across the 28 included studies; Table S3: Risk-of-bias appraisal—item-level judgements and supporting justifications; Table S4: Reports sought but not retrieved; Figure S1: Risk-of-bias appraisal of the 28 included studies, summarized by item and instrument.

## Abbreviations

The following abbreviations are used in this manuscript:

IC	Indirect Calorimetry
REE	Resting Energy Expenditure
ICU	Intensive Care Unit
PRISMA	Preferred Reporting Items for Systematic Reviews and Meta-Analyses
NA	Not applicable
SBS	Short Bowel Syndrome
PERT	Pancreatic Enzyme Replacement Therapy
cal	Calorie
kcal	Kilocalorie
kJ	Kilojoules
MJ	Megajoules
LCT	Long-chain triglycerides
MCT	Medium-chain triglycerides
AE	Absorption energy
FE	Fecal energy
DTA	Diagnostic Test Accuracy
NR	Not reported
SE	Standard error
EATL	Enteropathy-associated T-cell lymphoma
