# Supplementary material for: Measurement of Fecal Energy Loss Using Bomb Calorimetry in Adult Patient Populations: A Systematic Review and Meta-Analysis
Source: Nutrients. 2026 Sep 11;18(18):2976. doi: 10.3390/nu18182976 (PMC13610742; doi:10.3390/nu18182976)
Supplement: Supplementary file 1 [file nutrients-18-02976-s001.zip › nutrients-4499959-supplementary.pdf]

Supplementary Material

Measurement of Fecal Energy Loss Using Bomb Calorimetry in Adult Patient Populations: A Systematic Review and Meta-Analysis

Contents

Table S1. Search strategies for the original and revised searches, with the date each source was searched and notes on platform-specific syntax.

Table S2. Methodological reporting of stool bomb calorimetry across the 28 included studies.

Table S3. Risk-of-bias appraisal: item-level judgements and supporting justifications.

Table S4. Reports sought but not retrieved.

Figure S1. Risk-of-bias appraisal of the 28 included studies, summarized by item and instrument.

Supplementary Table S1. Search strategies

Original strategy — executed 8 September 2025

Source	String
PubMed	`("Adult"[Mesh] OR adult*) AND ("bomb calorimet*") AND ("energy absorption" OR "energy digestibility" OR "digestible energy" OR "fecal energy")`
Embase	`('adult'/exp OR adult*) AND ("bomb calorimet*") AND ("energy absorption" OR "energy digestibility" OR "digestible energy" OR "fecal energy")`
ClinicalTrials.gov, BASE, NDLTD	`("adult" OR "adults") AND ("bomb calorimetry" OR "bomb calorimeter") AND ("energy absorption" OR "energy digestibility" OR "digestible energy" OR "fecal energy")`

Limitations identified during peer review. All strings required the literal phrase "fecal energy" and contained no British-spelling variant, nor alternative terms such as "stool energy", "energy excretion" or "apparent digestibility". All strings also required an adult term, imposing a third restriction on retrieval. Together these reduced sensitivity, and an eligible study was subsequently identified that had been missed for this reason.

Revised strategy — executed 28 August 2026

Source	String
PubMed	`(bomb calorimet* OR "direct calorimetry" OR "ballistic bomb calorimeter" OR calorimet*) AND ("fecal" OR "faecal" OR "feces" OR "faeces" OR "stool" OR "stools" OR "intestinal") AND (energ* OR "absorption" OR "absorptiometry" OR digestib* OR "excretion" OR loss* OR "combustion") NOT (animals[Mesh] NOT humans[Mesh])`
Embase	`('bomb calorimetry':ti,ab,kw OR 'bomb calorimeter':ti,ab,kw OR 'ballistic bomb calorimeter':ti,ab,kw OR 'direct calorimetry':ti,ab,kw OR 'energy absorptiometry':ti,ab,kw OR calorimet*:ti,ab,kw) AND (fecal:ti,ab,kw OR faecal:ti,ab,kw OR feces:ti,ab,kw OR faeces:ti,ab,kw OR stool:ti,ab,kw OR stools:ti,ab,kw) AND (energ*:ti,ab,kw OR absorp*:ti,ab,kw OR digestib*:ti,ab,kw OR excret*:ti,ab,kw OR loss*:ti,ab,kw)`
ClinicalTrials.gov, NDLTD	`("bomb calorimetry" OR "bomb calorimeter" OR "direct calorimetry" OR "ballistic bomb calorimeter" OR "calorimetry" OR "calorimeter" OR "absorptiometry") AND ("fecal" OR "faecal" OR "feces" OR "faeces" OR "stool" OR "stools") AND ("energy" OR "energetic" OR "absorption" OR "absorptiometry" OR "digestible" OR "digestibility" OR "excretion" OR "loss" OR "losses" OR "combustion") NOT ("animals" NOT "humans")`
BASE	`("bomb calorimetry" OR "bomb calorimeter" OR "ballistic bomb calorimeter" OR "direct calorimetry" OR "energy absorptiometry" OR "calorimetry" OR "calorimeter") AND (fecal OR faecal OR feces OR faeces OR stool OR stools) AND (energy OR absorption OR digestibility OR excretion OR loss OR losses)`

No restriction on publication year was applied to either strategy. In accordance with the registered protocol, studies published in English, Dutch, Spanish or French were eligible.

Records retrieved, revised strategy

Source	Records
PubMed	291
Embase	416
ClinicalTrials.gov	136

Source	Records
BASE	713
NDLTD	91
**Total**	**1,647**
Duplicates removed before screening	644
Records screened	1,003

Platform notes

Search syntax is not portable between platforms, and the strings above differ accordingly.

Source	Note
PubMed	Automatic Term Mapping applied to untruncated terms. The Cochrane animal-exclusion filter `NOT (animals[Mesh] NOT humans[Mesh])` removes animal-only records without discarding human studies indexed under both terms.
Embase	Emtree terms with `:ti,ab,kw` field tags; truncation supported.
ClinicalTrials.gov	Free text only; no controlled vocabulary or field tags available.
BASE	Free text only. Truncation (`*`) is not supported, so word forms were enumerated in full.
NDLTD	Free text only; no controlled vocabulary available.

Verification. Before screening, the revised PubMed strategy was checked against the studies already included in the review to confirm that all were retrieved.

Supplementary Table S2. Methodological reporting of stool bomb calorimetry across included studies.

Each of the 28 included studies, listed alphabetically by first author, was assessed against a structured framework of methodological reporting elements derived from the procedural standards described by Basolo et al. The final column records study-specific observations explaining how each assessment was reached. NR = not reported.

Study	Intake measured concurrently	Collection duration	Collection completeness marker	Stool handling/ storage	Homogenization	Drying	Bomb calorimeter specified	Calibration	Replicates	Units clear	Notes
Achour, 2007	Yes, dietary intake quantified during the metabolic assessment using French Food Composition Tables	24 hours	NR	Stools collected during final 24h, weighed/pooled and stored frozen at -20°C	Yes	Yes	Bomb calorimetry reported; instrument/model NR	NR	NR	Yes	Participants were admitted for 3 days and remained on their usual diet. Dietary energy intake was calculated rather than directly measured. Stool was collected during the final 24h; no objective marker of collection completeness was reported.
Andreae, 2026	No, habitual intake assessed with a validated 1-month FFQ, not during a concurrent fecal balance period	Single stool sample, collection duration NR	NR	Stool sample returned to clinic; wet weight obtained, freeze-dried, ground and stored at -80°C until analysis	Ground after freeze-drying; homogenization not explicitly described	Yes	Yes -Parr 6200 oxygen bomb calorimeter	NR	NR	No	Dietary intake was estimated using validated Block 2014.1 1-month FFQ and was not synchronized with stool collection. Fecal energy was measured on 2.2g of freeze-dried, ground stool using a Parr 6200 bomb calorimeter. The Methods state that calories were calculated per gram of feces, whereas the Results report bomb calorimetry values only as "kcal" (~5300-5500), making the intended unit unclear and physiologically implausible if interpreted literally.
Atia, 2011	Yes, standardized meals provided during the same 72-hour metabolic-balance period as stool collection, duplicate meals were prepared, corrected for leftovers, weighed and chemically analyzed	72 hours	NR	24-hour stool collections frozen at -70°C as collected, thawed overnight, pooled over 72h, homogenized, aliquoted and refrozen at -70°C	Yes	No, bomb calorimetry performed on frozen wet specimens	Bomb calorimetry reported; instrument/model NR	NR	NR	Yes	A 72-hour metabolic-balance protocol was performed with concurrent standardized duplicate-diet assessment and complete stool collection. Actual food consumption, leftovers, emesis and fecal output were accounted for, and energy absorption was calculated as (intake - output) / intake * 100%. Stool was pooled and homogenized before bomb calorimetry. No objective fecal completeness marker, calorimeter model, calibration procedure or replicate bomb-calorimetry measurements were reported. Energy analysis was performed on wet specimens rather than lyophilized samples.
Brownell, 2020	Yes, 3-day weighed food records collected during the same 72-hour stool collection period	72 hours	NR	72-hour home stool collection, samples homogenized in bags with BagMixer, aliquot taken for bomb calorimetry	Yes	NR	Bomb calorimetry reported; instrument/model NR	NR	NR	Yes	Baseline fecal energy was assessed from a 72-hour home stool collection. Dietary intake was recorded concurrently using 3-day weighed food records. Stool samples were homogenized before aliquoting for bomb calorimetry. Fecal energy was reported as cal/g stool. No objective stool-collection completeness marker, drying procedure, calorimeter model, calibration procedure, or replicate bomb calorimetry measurements were reported.
Eliasson, 2022	Yes, duplicate dietary intake collected during each 72-h balance period	72 hours	NR	24-hour stool collections during inpatient metabolic balance study; weighed and processed to dry matter	NR	Yes	Bomb calorimetry reported; instrument/model NR	NR	NR	Yes (kJ/day)	Standardized 72-h inpatient metabolic balance studies were performed at baseline and post-treatment. Energy was measured by bomb calorimetry, technical details on homogenization, instrument model, calibration, and replicate measurements were not reported.
Erchinger, 2018	No, dietary intake was recorded during the 3 days preceeding each 3-day stool collection.	72 hours	NR	Stool weighed, diluted with water 2:1, homogenized to slurry, aliquoted and stored frozen until analysis	Yes	Yes	Yes - IKA C6000 global standards calorimeter with C6010 decomposition vessel	NR	NR	Yes	3-day food registration preceeded the 72-h stool collection rather than occuring concurrently. CEA was calculated from estimated energy intake and measured fecal energy loss. Technical calorimeter procedures were described in detail, but calibration or replicate measurements were not reported.
Haderslev, 2003	Yes, duplicate of unrestricted diet collected during the same 48-hour balance period	48 hours	NR	Diet and feces placed on ice, immediately frozen and stored at -20°C	Yes	Yes	Yes - adiabatic calorimeter, model C4000 A (IKA)	NR	NR	Yes	48-hour balance study with concurrent duplicate diet and fecal collection. Energy intake and fecal energy were both measured by bomb calorimetry. Calibration and replicate measurements were not reported.
Heydorn, 1999	Yes, duplicate diet collected during the same balance period	72 hours	NR	Stools collected on ice, immediately frozen at -20°C, 24-hour pooled samples analyzed	Yes	Yes	Yes - adiabatic calorimeter C4000 A (IKA)	NR	NR	Yes	Controlled isocaloric diet with duplicate-diet collection. Stool and urine were collected during the final 3 days of each condition. Dietary and fecal energy were measured by bomb calorimetry.
Heymsfield, 1981	Yes, intake measured during the same 5-day metabolic-balance period	5 days	Yes, stool collection began and ended with a distilled-water enema	5-day pooled stool collection, stored at -4°C, diluted en blended, aliquot stored at -20°C	Yes	Yes	Yes - Parr adiabatic oxygen bomb calorimeter, model 1341	NR	NR	Yes	Controlled 5-day metabolic-balance study after a 3-day equilibration period. Dietary intake was quantified and periodically checked by bomb calorimetry. Stool collection completeness was supported by enemas at the start and end of the collection period.
Jeppesen, 1998	Yes, duplicate diet collected during the same 3-day test periods	72h per diet condition	NR	Stools collected on ice, immediately frozen at -20°C, 24-hour pooled samples	Yes	Yes	Yes - adiabatic calorimeter C4000 A (IKA)	NR	NR	Yes	Randomized crossover with two controlled high-fat diets. Duplicate diets and fecal

				analyzed							samples were analyzed by bomb calorimetry. Review uses descriptive fecal and absorbed energy outcomes.
Jeppesen, 2000	Yes, duplicate diet collected during the same 48-hour balance period	48 hours	NR	Stools collected on ice, immediately frozen and stored at -20°C	Yes	Yes	Yes - adiabatic calorimeter C4000 A (IKA)	NR	NR	Yes	48-h free-choice metabolic-balance study with duplicate oral intake and fecal collection. Energy absorption was calculated as ingested minus excreted energy. Briljant blue was used to calculate intestinal transit before fecal collection.
Joly, 2009	Yes, intake measured during the same 3-day metabolic balance period	72 hours	NR	Stool/stoma output collected on ice and weighed daily during the 3-day metabolic period	NR	NR	Yes - Parr 1351 bomb calorimeter	NR	NR	Yes	Three-day controlled metabolic-balance period. Oral intake was quantified under dietitian supervision. Fecal energy was measured by bomb calorimetry and net intestinal absorption was calcultaed as the proportion of ingested energy not recovered in stool.
Mathur, 2016	Yes, controlled diet consumed during the same 3-day balance period	3 days	Yes, non-absorbable blue food dye used to delimit stool collection	All stool collected from first to second dye appearance and stored at -20°C	Yes	Yes	Yes - C5000 bomb calorimeter (IKA)	NR	NR	Yes	Individualized weight-maintaining diet over 3 days. Duplicate meal set prepared for calorimetry; leftovers were returned and analyzed. Stool collection was delimited by non-absorbable blue dye.
Messing, 1991	Yes, duplicate diet and actual intake assessed during the same 3-day metabolic-balance period	3 days	NR	Stools collected daily on ice and immediately frozen at -20°C; 3-day pooled samples	Yes	NR	Bomb calorimetry reported; instrument/model NR	NR	NR	Yes	Transit time defined by red carmine for calculation of equilibration period, before the collection period. 3-day metabolic-balance study after a 2-3 day equilibration period. Diet was prepared in duplicate and actual intake accounted for dish losses. Total caloric intake and fecal energy were measured by bomb calorimetry.
Naimi, 2019	Yes, exact replicas of dietary intake collected during the same 72 hours	72 hours per metabolic-balance period	NR	Fecal output collected in 24h buckets; contents weighed and processed for analysis	NR	Yes	Bomb calorimetry reported; instrument/model NR	NR	NR	Yes	Four 72h metabolic balance studies; exact replicas of dietary intake and feces collected. Only treatment-related changes in energy absorption are reported.
Nielsen, 1995	Yes, weighed intake and duplicate food portions were analyzed during the same balance periods	3x 3-day balance periods (beginning, middle and end of study)	NR	Feces collected during each 3-day balance period; further handling NR	NR	NR	Bomb calorimetry reported; instrument/model NR	NR	NR	Yes	Energy content of duplicate food and feces measured by bomb calorimetry in n=5. Reported fecal energy reflects repeated balance periods rather than a clearly identifiable baseline period.
Nordgaard, 1994	Yes, standardized study diets were provided during the same balance periods	3 days per diet period	NR	Stools collected daily, immediately frozen at -20°C, then pooled over each 3-day period	Yes	Yes	Yes - IKA C4000A adiabatic calorimeter	NR	NR	Yes	Patients first received a standardized 40:40 carb:fat reference diet. Two of initially 16 enrolled patients were excluded beacuse fecal energy loss was <2,1 MJ/day. Review-relevant estimate was derived from the 40:40 reference condition.
Peters, 2007	Yes, 4-day nutritional diary, with stool collection during the final 72h	72 hours	NR	All feces collected during the final 72h, weighed, homogenized and stored at -20°C	Yes	Yes	Yes - Gallenkamp ballistic bomb calorimeter, type CBB-33	NR	NR	Yes	Energy intake was estimated from a 4-day nutritional diary. Stool was collected during the last 72h. Intestinal energy absorption was calculated from energy intake minus fecal energy loss.
Pinar, 2025	Yes, duplicate portions of all consumed food and fluids were collected during the same 48 hours	48 hours	NR	All fecal output collected during the 48h; further sample handling details not clearly reported	NR	NR	Yes - C6000 calorimeter (IKA)	NR	NR	Yes	Two 48-hour metabolic-balance studies were performed, at baseline and week 24. During each, all fecal output and duplicate portions of all consumed foods and fluids were collected. Intestinal energy absorption was defined as oral intake minus fecal output.
Qian, 2024	No, relative energy content (REC) used dietary intake from the previous day, obtained by ASA24 recall	Single stool sample, no defined multi-day fecal collection	NR/NA - no complete fecal collection was performed	Fecal samples collected at each study time point and stored at -80°C until analysis	NR	NR	Yes - Parr 6200 Calorimeter	NR	NR	Partially, fecal energy density and REC were described, but no directly extractable numerical REC summary was reported	REC was calculated from the energy content of a fecal sample divided by self-reported dietary energy intake from the previous day. No complete fecal collection or fecal weight was available. Numerical REC results were presented graphically but no directly extractable summary value was reported.
Strack van Schijndel, 2006	Yes, EN was provided continuously during the fecal collection period	24-72 hours	No - no marker used, fecal leakage was estimated	Feces collected with a feces-collector, taken daily to the laboratory, homogenized, cooled, then pooled and re-homogenized	Yes	Yes	Yes - Gallenkamp ballistic bomb calorimeter, type CBB-33	NR	NR	Yes	Enteral intake was calculated from the declared composition of the feeding formulas rather than measured by duplicate-diet calorimetry. Fecal collection lasted 24-72h; leakage was estimated when it occured.
Vaisman, 2006	Yes, 3-day food records completed during the same 3-day stool collection period	3 days	Yes, activated charcoal given at the beginning of days 1 and 4 to delimit stool collection	All stools collected for 3 days and stored at -20°C until analysis	NR	Yes	Yes - Parr ballistic bomb calorimeter	NR	NR	Yes	Three-day food records and stool collection were performed concurrently. Dietary intake was estimated from food-composition tables rather than measured bu duplicate-diet calorimetry. Stool collection was delimited using activated charcoal.
Verbiest, 2024	Yes, oral intake assessed during the same 72 hours	72 hours	NR	Detailed sample handling reffered to previous methodology	NR	NR	Bomb calorimetry reported; instrument/model NR	NR	NR	Yes	72h metabolic balance study during a 5-day admission at baseline, 4 and 48 weeks.
Verbiest, 2025	Yes, duplicate portions of all consumed food and fluids were collected during the same 72 hours	72 hours	NR	Feces stored frozen during collection, then thawed, weighed, homogenized, aliquoted and stored at -80°C	Yes	Yes	Yes - C6000 calorimeter (IKA)	Yes	Yes	Yes	Full 72-h metabolic-balance study with duplicate oral intake, fecal and urinary collection. Samples were processed in both Copenhagen and Leuven to assess analytical and process transferability. Energy measurements showed good inter-laboratory agreement.
Wierdsma, 2011	Yes, enteral intake was continuously during the same	72 hours	No - no marker used, collections with more than	Feces collected in preweight buckets/flexiseal, weighed,	Yes	Yes	Yes - Gallenkamp ballistic bomb calorimeter, type CBB-33	NR	NR	Yes	Actual energy intake was calculated from digitally

	72-h collection period		minimal fecal leakage were excluded	homogenized and immediately stored at <4°C until analysis							recorded feeding volumes and formula composition rather than duplicate-diet calorimetry. Stool collection completeness was monitored by nurses; collections with more than minimal leakage were excluded.
Wierdsma, 2016	Yes, 4-day nutritional diary, with stool collection during days 2-4	72 hours	NR	All stools collected in 5l buckets, kept cool/dry, weighed on arrival, homogenized and stored at <4°C until analysis	Yes	Yes	Yes - Gallenkamp ballistic bomb calorimeter, type CBB-33	NR	NR	Yes	Dietary intake was estimated from a 4-day weighed food diary and NEVO food-composition data rather than duplicate-diet calorimetry. Stool was collected during days 2-4 of the same study period.
Witvliet-van Nierop, 2017	Yes, 4-day nutritional diary, with stool collection during days 2-4	72 hours	NR	All stools collected in 5l buckets, kept cool/dry, weighed on arrival, homogenized and stored at <4°C until analysis	Yes	Yes	Yes - Gallenkamp ballistic bomb calorimeter, type CBB-33	NR	NR	Yes	Dietary intake was estimated from a 4-day weighed food diary and NEVO food-composition data rather than duplicate-diet calorimetry. Stool was collected during days 2-4 of the same study period. One of 16 participants did not complete fecal collection, so energy outcomes were available for n = 15.
Woolf, 1983	Yes, controlled diet and duplicate 24h diet analyzed concurrently	4 consecutive 24h collections per diet	Yes	Complete 24h stool/stoma collections; 50 ml stool sample from each collection used for calorimetry	NR	Yes	Yes - Gallenkamp ballistic bomb calorimeter	NR	NR	Yes	Both dietary and fecal energy were measured by bomb calorimetry. Strong collection control but mean absorption reported without statistical spread.

Summary of reporting completeness (n = 28 studies)

**Reporting element**	**Studies reporting**
Energy intake quantified concurrently with stool collection	25/28
Calorimeter make and model identified	20/28
Drying procedure described	20/28
Homogenization described	18/28
Marker or procedure for collection completeness	4/28
Calibration procedure reported	1/28
Replicate determinations reported	1/28
Units of measurement stated unambiguously	26/28

Supplementary Table S3. Risk-of-bias appraisal: item-level judgements and justifications.

Studies are listed alphabetically by first author within each instrument. Instruments were selected to match the design of the data used in this review rather than the design of the parent study. Each study was appraised against the specific outcome used here, which is stated for every study. Appraisals were performed independently by two reviewers (E.V. and M.A.); agreement was complete and no discrepancies required resolution. NA = not applicable.

JBI for Analytical Cross-sectional Studies (2026) (n = 21)

Study	Design	Review outcome assessed	Item	Judgement / justification
**Achour 2007**	Observational comparative metabolic balance study	Apparent energy digestibility / intestinal energy absorption	Q1. Inclusion criteria clearly defined?	Unclear. Clear description of participants but no inclusion- or exclusion criteria reported
			Q2. Objective, standard criteria for measurement of condition?	Yes. SBS was based on documented extensive intestinal resection, with remaining bowel anatomy reported for each patient.
			Q3. Exposure measured in a valid and reliable way?	NA
			Q4. Outcomes measured in a valid and reliable way?	Unclear. Calculated from quantified dietary intake and fecal energy measured through bomb calorimetry. However, the completeness of the 24-hour fecal collection was not objectively verified, and limited information was provided on technical quality-control procedures for bomb calorimetry.
			Q5. Confounding factors identified?	NA. Not applicable to the result used in this review, as the reviewer did not use the comparison between patients with SBS and healthy volunteers to estimate an exposure-outcome association.
			Q6. Strategies to deal with confounders stated?	NA. Not applicable to the result used in this review, as only the descriptive energy absorption estimate from patients with SBS was extracted; no exposure-outcome association or comparison with healthy controls contributed to the review outcome
			Q7. Appropriate statistical analysis?	Yes. Apparent energy digestibility was calculated at the participant level and summarized as a group mean with SEM. Appropriate non-parametric statistical tests were used for the analyses reported in the study.
			Q8. Study subjects and setting described in detail?	Yes. The study populations were described in sufficient detail, including age, sex, underlying diagnosis and residual intestinal anatomy, and the metabolic study was conducted during a three-day admission at the Clinical Center.
**Andreae 2026**	Cross-sectional observational comparative study	Energy intake and fecal energy	Q1. Inclusion criteria clearly defined?	Unclear. HIV-positive men aged 18-60 years were recruited from an infectious disease specialty clinic, but explicit eligibility and exclusion criteria for participant inclusion were not fully reported.
			Q2. Objective, standard criteria for measurement of condition?	Yes. HIV-positive status was based on an established clinical diagnosis in patients recruited from an infectious diseases specialty clinic and receiving antiretroviral therapy.
			Q3. Exposure measured in a valid and reliable way?	NA. Not applicable to the result used in this review, as only the descriptive fecal energy measurement and intake energy from HIV-positive participants was considered and no exposure-outcome association was evaluated.
			Q4. Outcomes measured in a valid and reliable way?	Yes + Unclear. Energy intake was assessed using the validated Block 2014.1 one-month Food Frequency Questionnaire, with dietary data processed by NutritionQuest. Fecal energy was objectively measured using bomb calorimetry on freeze-dried, ground stool samples with a specified Parr 6200 calorimeter. However, the reported units are internally inconsistent: the Methods state that energy was calculated per gram of feces, whereas the Results report values of approximately 5300-5500 "kcal", which are physiologically implausible if interpreted literally. The intended unit cannot therefore be determined with confidence, and calibration and replicate bomb calorimetry measurements were not clearly reported.
			Q5. Confounding factors identified?	NA. Not applicable to the results used in this review, as only descriptive energy intake and fecal energy measurements from the HIV-positive group were extracted, no exposure-outcome association or between-group effect estimate contributed to the review outcome.
			Q6. Strategies to deal with confounders stated?	NA. Not applicable to the results used in this review, as no exposure-outcome association or between-group effect estimate was extracted; therefore, strategies to control for confounding were not required for the review-relevant descriptive outcomes.
			Q7. Appropriate statistical analysis?	Yes. Energy intake was summarized using mean and standard deviation, and between-group comparisons were performed using Welch's t-test, which was appropriate for the independent groups. Fecal energy values were summarized using mean and standard deviation, and between-group comparisons were performed using Welch's t-test, an appropriate method for comparing two independent groups.
			Q8. Study subjects and setting described in detail?	Yes. The study population and setting were described in sufficient detail, including participant age range, HIV status, recruitment setting, antiretroviral treatment, and relevant demographic and clinical characteriztics.
**Brownell 2020**	Open-label cohort study; baseline cross-sectional component used	Fecal energy	Q1. Inclusion criteria clearly defined?	Yes. Eligibility criteria were clearly defined, including age range, established chronic pancreatitis, risk indicators for fat malabsorption, stable health status, and explicit exclusion criteria.
			Q2. Objective, standard criteria for measurement of condition?	Yes. Chronic pancreatitis was diagnosed by a gastroenterologist using criteria recommended by the American Pancreatic Association Practice Guidelines.
			Q3. Exposure measured in a valid and reliable way?	NA. Not applicable to the result used in this review, as only the descriptive baseline fecal energy measurement from patients with chronic pancreatitis was extracted and no exposure-outcome association was evaluated.
			Q4. Outcomes measured in a valid and reliable way?	Unclear. Fecal energy was measured from a 72-hour stool collection that was homogenized before aliquoting for bomb calorimetry. However, no objective marker of collection completeness was reported, and limited information was provided on the bomb calorimeter, calibration procedures, or replicate measurements.
			Q5. Confounding factors identified?	NA. Not applicable to the result used in this review, as only the descriptive baseline fecal energy measurement from the CP group was extracted; therefore, adjustment for confounding was not required for the review-relevant descriptive outcome.
			Q6. Strategies to deal with confounders stated?	NA. Same as previous
			Q7. Appropriate statistical analysis?	Yes. Fecal energy was summarized using mean and standard deviation. Between-group baseline comparisons were performed using an unpaired two-sample t-test, which was appropriate for the independent study groups.
			Q8. Study subjects and setting described in detail?	Yes. The study population and setting were described in sufficient detail, including recruitment source, clinical setting, age range, CP diagnostic criteria, eligibility criteria, and relevant demographic and clinical characteriztics.
**Erchinger 2018**	Non-randomized controlled trial; baseline cross-sectional component used	Coefficient of energy absorption	Q1. Inclusion criteria clearly defined?	Yes. Eligibility criteria were clearly defined, including confirmed chronic pancreatitis, pancreatic exocrine insufficiency, and relevant exclusion criteria.
			Q2. Objective, standard criteria for measurement of condition?	Yes. Chronic pancreatitis and pancreatic exocrine insufficiency were defined using established clinical criteria and fecal elastase-1 testing.
			Q3. Exposure measured in a valid and reliable way?	NA. Not applicable to the result used in this review, only descriptive baseline coefficient of energy absorption were used.
			Q4. Outcomes measured in a valid and reliable way?	Unclear. CEA was calculated from measured fecal energy loss and documented dietary energy intake; however, dietary intake was recorded during the three days preceeding rather than during the stool collection period.
			Q5. Confounding factors identified?	NA. Not applicable to the result used in this review, as only the descriptive baseline CEA was used.
			Q6. Strategies to deal with confounders stated?	NA. Same as previous.
			Q7. Appropriate statistical analysis?	Yes. CEA was reported as mean with standard deviation, and statistical tests were selected according to data distribution.
			Q8. Study subjects and setting described in detail?	Yes. The study population and setting were described in sufficient detail, including diagnostic criteria and key demographic characteriztics.
**Haderslev 2003**	Cross-sectional observational study	Intestinal energy absorption	Q1. Inclusion criteria clearly defined?	Yes. Eligibility criteria were clearly defined, including criteria related to intestinal resection, energy malabsorption, and relevant exclusions.
			Q2. Objective, standard criteria for measurement of condition?	Yes. Patient status was defined using objective clinical and anatomical criteria, including documented intestinal resection and fecal energy loss.
			Q3. Exposure measured in a valid and reliable way?	NA. Not applicable, only the descriptive intestinal energy absorption result was used.

			Q4. Outcomes measured in a valid and reliable way?	Yes. Energy absorption was measured using a 48-hour balance study with duplicate dietary collection and bomb calorimetry of both diet and feces.
			Q5. Confounding factors identified?	NA. Not applicable, only the descriptive intestinal energy absorption result was used.
			Q6. Strategies to deal with confounders stated?	NA. Same as previous.
			Q7. Appropriate statistical analysis?	Yes. Energy absorption was appropriately summarized as mean and standard deviation, with statistical methods suitable for the study analyses.
			Q8. Study subjects and setting described in detail?	Yes. The study population and setting were described in sufficient detail, including demographic and intestinal anatomical characteriztics.
**Heydorn 1999**	Randomized cross-over trial	Intestinal energy absorption	Q1. Inclusion criteria clearly defined?	Yes. Eligibility criteria were clearly defined.
			Q2. Objective, standard criteria for measurement of condition?	Yes. SBS was clearly characterized using documented intestinal resection and objective clinical criteria.
			Q3. Exposure measured in a valid and reliable way?	NA. Not applicable, only the descriptive no-treatment intestinal energy absorption result was used.
			Q4. Outcomes measured in a valid and reliable way?	Yes. Energy absorption was measured using a controlled metabolic-balance protocol with duplicate diets and bomb calorimetry of dietary and fecal energy.
			Q5. Confounding factors identified?	NA. Not applicable, only the descriptive intestinal energy absorption result was used.
			Q6. Strategies to deal with confounders stated?	NA. Same as previous.
			Q7. Appropriate statistical analysis?	Yes. Energy absorption was appropriately summarized as mean and standard deviation, with statistical methods suitable for the study design.
			Q8. Study subjects and setting described in detail?	Yes. Participants were described in sufficient clinical and anatomical detail.
**Heymsfield 1981**	Cross-sectional observational study	Energy digestibility	Q1. Inclusion criteria clearly defined?	Unclear. Participants were described, but explicit eligibility criteria for study entry were not clearly reported.
			Q2. Objective, standard criteria for measurement of condition?	Unclear. Malabsorption was classified based on a history of diarrhea or steatorrhea rather than clearly defined standardized diagnostic criteria.
			Q3. Exposure measured in a valid and reliable way?	NA. Not applicable, only the descriptive intestinal energy absorption result was used.
			Q4. Outcomes measured in a valid and reliable way?	Yes. Energy absorption was assessed during a controlled metabolic-balance period using measured dietary intake and fecal energy determined by bomb calorimetry.
			Q5. Confounding factors identified?	NA. Not applicable, only the descriptive intestinal energy absorption result was used.
			Q6. Strategies to deal with confounders stated?	NA. Same as previous.
			Q7. Appropriate statistical analysis?	Yes. Energy digestibility was appropriately reported using descriptive statistics, including mean, SD and range
			Q8. Study subjects and setting described in detail?	Yes. The clinical setting, patient groups and underlying diagnoses were adequately described.
**Jeppesen 1998**	Randomized cross-over trial	Fecal energy and absorbed energy	Q1. Inclusion criteria clearly defined?	Yes. Eligibility criteria were clearly described.
			Q2. Objective, standard criteria for measurement of condition?	Yes. Patients were characterized using documented small-bowel resection and fecal loss criteria.
			Q3. Exposure measured in a valid and reliable way?	NA. Not applicable, only the descriptive energy absorption and fecal energy outcomes were used.
			Q4. Outcomes measured in a valid and reliable way?	Yes. Energy outcomes were assessed using controlled metabolic-balance periods with duplicate diets and bomb calorimetry of diet and feces.
			Q5. Confounding factors identified?	NA. Not applicable, only descriptive outcomes were used.
			Q6. Strategies to deal with confounders stated?	NA. Same as previous.
			Q7. Appropriate statistical analysis?	Yes. Energy outcomes were reported using mean and standard deviation, with statistical methods appropriate to the crossover design.
			Q8. Study subjects and setting described in detail?	Yes. Participants were described in sufficient demographic and intestinal anatomical detail.
**Jeppesen 2000**	Cross-sectional observational study	Energy absorption	Q1. Inclusion criteria clearly defined?	Yes. Eligibility criteria for the HPN and non-HPN patient groups were clearly described.
			Q2. Objective, standard criteria for measurement of condition?	Yes. Intestinal insufficiency/failure was defined using HPN dependence, documented intestinal anatomy and/or previously measured fecal energy loss.
			Q3. Exposure measured in a valid and reliable way?	NA. Not applicable, only descriptive energy outcomes were used.
			Q4. Outcomes measured in a valid and reliable way?	Yes. Energy absorption was measured during a 48-hour balance study with duplicate dietary collection and bomb calorimetry.
			Q5. Confounding factors identified?	NA. Not applicable, only descriptive outcomes were used.
			Q6. Strategies to deal with confounders stated?	NA. Same as previous.
			Q7. Appropriate statistical analysis?	Yes. Energy outcomes were summarized using median and interquartile range, with appropriate non-parametric analyses.
			Q8. Study subjects and setting described in detail?	Yes. The patient groups were described in sufficient demographic, diagnostic and intestinal anatomical detail.
**Joly 2009**	Randomized cross-over trial	Energy absorption	Q1. Inclusion criteria clearly defined?	Yes. Eligibility criteria were clearly defined.
			Q2. Objective, standard criteria for measurement of condition?	Yes. SBS was defined using documented extensive small-bowel resection and clinical criteria.
			Q3. Exposure measured in a valid and reliable way?	NA. Not applicable, only the descriptive energy absorption was used.
			Q4. Outcomes measured in a valid and reliable way?	Yes. Energy absorption was measured during a controlled 3-day metabolic-balance period using measured intake and bomb calorimetry of fecal energy.
			Q5. Confounding factors identified?	NA. Not applicable, only descriptive energy absorption was used.
			Q6. Strategies to deal with confounders stated?	NA. Same as previous.
			Q7. Appropriate statistical analysis?	Yes. Energy absorption was appropriately summarized as mean and standard deviation, with paired non-parametric analyses for the crossover comparisons.
			Q8. Study subjects and setting described in detail?	Yes. Participants and the tertiary intestinal-failure setting were described in sufficient clinical and anatomical detail.
**Messing 1991**	Cross-sectional observational study	Net intestinal energy absorption	Q1. Inclusion criteria clearly defined?	Yes. Eligibility criteria were clearly defined.
			Q2. Objective, standard criteria for measurement of condition?	Yes. SBS was characterized using documented extensive bowel resection and residual intestinal anatomy.
			Q3. Exposure measured in a valid and reliable way?	NA. Not applicable, only the descriptive energy absorption was used.
			Q4. Outcomes measured in a valid and reliable way?	Yes. Energy absorption was measured during a controlled metabolic-balance period using measured dietary intake and fecal energy determined by bomb calorimetry.
			Q5. Confounding factors identified?	NA. Not applicable, only descriptive energy absorption was used.
			Q6. Strategies to deal with confounders stated?	NA. Same as previous.
			Q7. Appropriate statistical analysis?	Yes. Energy absorption was appropriately summarized as mean and standard deviation.
			Q8. Study subjects and setting described in detail?	Yes. Participants were described in sufficient clinical and anatomical detail.
**Nordgaard 1994**	Randomized cross-over trial; cross-sectional baseline result used	Energy absorbed	Q1. Inclusion criteria clearly defined?	Yes. Eligibility and exclusion were clearly described; however, two patients were excluded because fecal energy loss was <2.1 MJ/day. This introduces a selection concern for the descriptive absorption estimate.
			Q2. Objective, standard criteria for measurement of condition?	Yes. Short bowel anatomy and colon continuity were clearly documented.
			Q3. Exposure measured in a valid and reliable way?	NA. Not applicable, only energy absorption was used.
			Q4. Outcomes measured in a valid and reliable way?	Yes. Dietary and fecal energy were measured during controlled balance periods using bomb calorimetry.
			Q5. Confounding factors identified?	NA. Not applicable, only descriptive outcome was used.
			Q6. Strategies to deal with confounders stated?	NA. Same as previous.
			Q7. Appropriate statistical analysis?	Yes. Appropriate descriptive and paired analyses were used.
			Q8. Study subjects and setting described in detail?	Yes. Patient anatomy and study conditions were described in detail.
**Peters 2007**	DTA study, cross-sectional descriptive component used	Intestinal energy absorption capacity	Q1. Inclusion criteria clearly defined?	Yes. Eligibility criteria were clearly defined for each patient group.
			Q2. Objective, standard criteria for measurement of condition?	Yes. CeD, RCeD and SBS were defined using objective diagnostic, histological and/or anatomical criteria.
			Q3. Exposure measured in a valid and reliable way?	NA. Not applicable, only the descriptive intestinal energy absorption capacity was used.

			Q4. Outcomes measured in a valid and reliable way?	Unclear. Intestinal energy absorption capacity was assessed using a 4-day dietary record, 72-hour fecal collection and bomb calorimetry.
			Q5. Confounding factors identified?	NA. Not applicable, only descriptive intestinal energy absorption capacity was used.
			Q6. Strategies to deal with confounders stated?	NA. Same as previous.
			Q7. Appropriate statistical analysis?	Yes. Intestinal energy absorption capacity was appropriately reported as mean and standard deviation.
			Q8. Study subjects and setting described in detail?	Yes. Patient groups and the tertiary clinical setting were described in sufficient detail.
**Qian 2024**	Prospective cohort study	Relative fecal energy content	Q1. Inclusion criteria clearly defined?	Unclear. Eligibility criteria were referred to a previous publication and were not fully reported in the present article.
			Q2. Objective, standard criteria for measurement of condition?	Yes. Participants were patients undergoing metabolic and bariatric surgery for severe obesity.
			Q3. Exposure measured in a valid and reliable way?	NA. Not applicable, only descriptive fecal energy/energy harvest measurements were used.
			Q4. Outcomes measured in a valid and reliable way?	Unclear. Fecal energy density was measured by bomb calorimetry, but relative energy content relied on self-reported dietary intake and a single fecal sample rather than complete fecal collection.
			Q5. Confounding factors identified?	NA. Not applicable, no exposure-outcome association was used in this review.
			Q6. Strategies to deal with confounders stated?	NA. Same as previous.
			Q7. Appropriate statistical analysis?	Yes. The energy measurements were appropriately summarized and analyzed.
			Q8. Study subjects and setting described in detail?	Yes. The study population, surgical procedures and recruitment from two bariatric clinics were adequately described.
**Strack van Schijndel 2006**	Prospective observational study	Mean total energy absorption	Q1. Inclusion criteria clearly defined?	Yes. Eligibility criteria were clearly defined for fully enterally fed, mechanically ventilated ICU patients with loose stools.
			Q2. Objective, standard criteria for measurement of condition?	Yes. ICU status, mechanical ventilation, enteral feeding and clinical stability were explicitly defined.
			Q3. Exposure measured in a valid and reliable way?	NA. Not applicable, only descriptive energy absorption was used.
			Q4. Outcomes measured in a valid and reliable way?	Unclear. Fecal energy was measured with bomb calorimetry, but enteral energy intake was calculated from formula composition rather than measured by duplicate-diet calorimetry, and some fecal losses were estimated after leakage.
			Q5. Confounding factors identified?	NA. Not applicable, only descriptive energy absorption was used.
			Q6. Strategies to deal with confounders stated?	NA. Same as previous.
			Q7. Appropriate statistical analysis?	Yes. Energy absorption was appropriately summarized using mean, standard deviation and range.
			Q8. Study subjects and setting described in detail?	Yes. The ICU setting and patient characteriztics were described in sufficient clinical detail.
**Vaisman 2006**	Cross-sectional observational study	Energy malabsorption percentage	Q1. Inclusion criteria clearly defined?	Yes. Eligibility criteria were clearly defined, including age, Crohn's disease remission, stable body weight and relevant exclusions.
			Q2. Objective, standard criteria for measurement of condition?	Yes. Crohn's disease was diagnosed using clinical, morphological and histopathological criteria, and remission was defined using a Crohn's Disease Activity Index <150.
			Q3. Exposure measured in a valid and reliable way?	NA. Not applicable, only descriptive energy malabsorption was used.
			Q4. Outcomes measured in a valid and reliable way?	Unclear. Fecal energy was measured with bomb calorimetry, but dietary energy intake was estimated from 3-day food records rather than duplicate-diet calorimetry.
			Q5. Confounding factors identified?	NA. Not applicable, only descriptive energy malabsorption percentage was used.
			Q6. Strategies to deal with confounders stated?	NA. Same as previous.
			Q7. Appropriate statistical analysis?	Yes. Energy malabsorption was appropriately summarized and group comparisons were adjusted or sex where relevant.
			Q8. Study subjects and setting described in detail?	Yes. The patient population and outpatient clinical setting were adequately described.
**Verbiest 2024**	Single-arm open label phase 2 trial; cross-sectional component assessed	Relative energy absorption	Q1. Inclusion criteria clearly defined?	Yes. Detailed inclusion and exclusion criteria for adults with SBS-IF and colon-in-continuity were predefined.
			Q2. Objective, standard criteria for measurement of condition?	Yes. SBS-IF was defined using remnant bowel anatomy, parenteral support requirements and objective baseline metabolic balance data.
			Q3. Exposure measured in a valid and reliable way?	NA. Not applicable, only relative energy absorption was used.
			Q4. Outcomes measured in a valid and reliable way?	Yes. Energy absorption was assessed during a standardized 72h metabolic-balance study with duplicate dietary intake and fecal collection, using bomb calorimetry.
			Q5. Confounding factors identified?	NA. Not applicable, only descriptive baseline outcome was used.
			Q6. Strategies to deal with confounders stated?	NA. Same as previous.
			Q7. Appropriate statistical analysis?	Yes. Relative energy absorption was appropriately summarized with the mean and 95% CI.
			Q8. Study subjects and setting described in detail?	Yes. Patient characteriztics, SBS anatomy, parenteral support and clinical setting were described in detail.
**Wierdsma 2011**	DTA study; cross-sectional descriptive component assessed	Energy absorption capacity	Q1. Inclusion criteria clearly defined?	Yes. Eligibility criteria were clearly defined for stable, fully enterally fed ICU patients.
			Q2. Objective, standard criteria for measurement of condition?	Yes. ICU status and clinical stability were defined using explicit respiratory, hemodynamic and feeding criteria.
			Q3. Exposure measured in a valid and reliable way?	NA. Not applicable, only descriptive energy absorption capacity was used.
			Q4. Outcomes measured in a valid and reliable way?	Unclear. Fecal energy was measured with bomb calorimetry, but energy intake was calculated from enteral formula composition rather than duplicate-diet calorimetry.
			Q5. Confounding factors identified?	NA. Not applicable, only descriptive energy absorption capacity was used.
			Q6. Strategies to deal with confounders stated?	NA. Same as previous.
			Q7. Appropriate statistical analysis?	Yes. Energy absorption capacity was appropriately summarized using the mean and standard deviation.
			Q8. Study subjects and setting described in detail?	Yes. The tertiary ICU setting and patient characteriztics were described in sufficient detail.
**Wierdsma 2016**	Cross-sectional observational study	Mean energy absorption	Q1. Inclusion criteria clearly defined?	Yes. Patients were consecutively recruited and the diagnostic groups were clearly defined.
			Q2. Objective, standard criteria for measurement of condition?	Yes. CD, RCDII and EATL were diagnosed using established histological, serological, immunophenotypic and WHO criteria.
			Q3. Exposure measured in a valid and reliable way?	NA. Not applicable, only mean energy absorption was used.
			Q4. Outcomes measured in a valid and reliable way?	Unclear. Fecal energy was measured with bomb calorimetry, but energy intake was estimated from a 4-day food diary rather than duplicate-diet calorimetry.
			Q5. Confounding factors identified?	NA. Not applicable, only descriptive energy absorption was used.
			Q6. Strategies to deal with confounders stated?	NA. Same as previous.
			Q7. Appropriate statistical analysis?	Yes. Mean energy absorption was appropriately summarized using mean and standard deviation and compared using parametric or non-parametric tests as appropriate.
			Q8. Study subjects and setting described in detail?	Yes. The tertiary-care setting and the three patient groups were described in sufficient clinical detail.
**Witvliet-van Nierop 2017**	Cross-sectional observational study	Energy absorption capacity	Q1. Inclusion criteria clearly defined?	Unclear. The study population was described, but detailed eligibility criteria for inclusion in the nutritional assessment were not fully reported in this article.
			Q2. Objective, standard criteria for measurement of condition?	Yes. Locally advanced pancreatic cancer was established within the PANFIRE study before experimental IRE treatment.
			Q3. Exposure measured in a valid and reliable way?	NA. Not applicable, only mean energy absorption capacity was used.
			Q4. Outcomes measured in a valid and reliable way?	Unclear. Fecal energy was measured with bomb calorimetry, but energy intake was estimated from a 4-day food diary rather than duplicate-diet calorimetry.
			Q5. Confounding factors identified?	NA. Not applicable, only descriptive energy absorption capacity was used.
			Q6. Strategies to deal with confounders stated?	NA. Same as previous.
			Q7. Appropriate statistical analysis?	Yes. Energy absorption capacity was appropriately summarized using median and interquartile range according to distribution.

			Q8. Study subjects and setting described in detail?	Yes. The outpatient clinical setting and patient characteriztics were described in sufficient detail.
**Woolf 1983**	Randomized cross-over trial; cross-sectional baseline result used	Energy absorption	Q1. Inclusion criteria clearly defined?	Yes. Patients had short bowel following intestinal resection and were clinically stable for at least six months.
			Q2. Objective, standard criteria for measurement of condition?	Yes. Remaining intestinal anatomy and clinical characteriztics were clearly documented.
			Q3. Exposure measured in a valid and reliable way?	NA. Not applicable, only relative energy absorption was used.
			Q4. Outcomes measured in a valid and reliable way?	Yes. Dietary intake was controlled, complete stool collections were performed, completeness was checked with a marker, and energy was measured using bomb calorimetry.
			Q5. Confounding factors identified?	NA. Not applicable, only descriptive baseline outcome was used.
			Q6. Strategies to deal with confounders stated?	NA. Same as previous.
			Q7. Appropriate statistical analysis?	Yes. Paired statistical testing was appropriate for the crossover design; however, no spread statistic is reported.
			Q8. Study subjects and setting described in detail?	Yes. Patient characteriztics, remaining bowel anatomy and study condition were described in detail.

JBI checklist for quasi-experimental studies (2026) (n = 5)

Study	Design	Review outcome assessed	Item	Judgement / justification
**Atia 2011**	Single arm trial	Mean energy absorption	Q1. Clear in study what is the cause and what is the effect?	Yes. The temporal sequence was clearly established: baseline metabolic balance measurements were completed before pectin supplementation was initiated, and the same assessments were repeated after the supplementation period.
			Q2. Control group?	No. No separate control group was included; the same six patients with SBS were assessed before and after pectin supplementation in a single-arm before-after design.
			Q3. Participants included in comparison similar?	Yes. The comparison was within the same six SBS patients before and after pectin supplementation, so participant characteriztics were inherently comparable across study periods.
			Q4. Participants included in comparison received similar care?	Yes. Participants received comparable care during both study periods, including the same standardized diet and continuation of usual antidiarrheal medication and PN; the principal difference between periods was the addition of pectin supplementation.
			Q5. Multiple measurements of outcome (pre and post intervention)	No. Intestinal energy absorption was measured once during the baseline metabolic-balance period and once after pectin supplementation; multiple repeated measurements were not performed both before and after the intervention.
			Q6. Outcomes of participants included in comparison measured the same way?	Yes. Intestinal energy absorption was measured using the same metabolic-balance procedures during the baseline and post-intervention periods, including comparable dietary assessment, fecal collection, bomb calorimetry, and the same calculation of net digestive absorption.
			Q7. Outcomes measured in a reliable way?	Yes. Intestinal energy absorption was measured using a structured 72-hour metabolic-balance protocol with complete stool collection, duplicate meals, standardized sample processing, bomb calorimetry, and a predefined calculation of net digestive absorption.
			Q8. Follow-up complete? If not, differences between groups (follow-up) adequately described and analyzed?	Yes. Data for the review-relevant baseline energy absorption outcomes were available for all six participants, both as a percentage and absolute energy absorption (kcal/day).
			Q9. Appropriate statistical analysis?	Yes. Energy absorption was summarized using mean and standard deviation, and the paired pre-post comparison was analyzed using an exact Wilcoxon signed-rank test, which was appropriate for the small, paired sample.
**Eliasson 2022**	Single arm trial	Absolute change in intestinal energy absorption	Q1. Clear in study what is the cause and what is the effect?	Yes. The temporal sequence was clearly established: a baseline 72-hour metabolic balance study was completed before apraglutide treatment, and the same assessment was repeated at the end of the 4-week treatment period.
			Q2. Control group?	No. No separate control group was included; all eight patients received apraglutide and were evaluated using a within-subject before-after design.
			Q3. Participants included in comparison similar?	Yes. The comparison was within the same eight patient before and after the intervention, so participant characteriztics were inherently comparable across study periods.
			Q4. Participants included in comparison received similar care?	Yes. Participants received comparable care during the baseline and post-treatment metabolic balance periods. Daily PN and dietary fluid intake were kept constant, and concomitant medications remained unchanged; the principal difference was apraglutide treatment.
			Q5. Multiple measurements of outcome (pre and post intervention)	No. Intestinal energy absorption was assessed using one 72-hour metabolic balance study at baseline and one at the end of treatment. The reported review-relevant result was the absolute change from baseline in energy absorption (kJ/day); multiple repeated measurements were not performed both before and after the intervention.
			Q6. Outcomes of participants included in comparison measured the same way?	Yes. Intestinal energy absorption was measured using the same 72-hour metabolic balance procedures at baseline and at the end of treatment, including duplicate dietary intake collection, fecal output collection, dry-matter processing, and bomb calorimetry.
			Q7. Outcomes measured in a reliable way?	Yes. Intestinal energy absorption was measured using structured 72-hour inpatient metabolic balance studies with duplicate dietary intake collection, complete fecal output collection, dry-matter processing, and bomb calorimetry. The same procedure was applied at baseline and post-treatment.
			Q8. Follow-up complete? If not, differences between groups (follow-up) adequately described and analyzed?	Yes. Follow-up was complete for the review-relevant outcome. All eight enrolled patients completed the trial and were included in the full analysis for the change in intestinal energy absorption.
			Q9. Appropriate statistical analysis?	Yes. The change in intestinal energy absorption from baseline to the end of treatment was analyzed using a paired student t-test, which was appropriate for the within-subject design. Estimates were reported with 95% confidence intervals and p-values, and nonparametric analyses yielded comparable results.
**Mathur 2016**	Single arm trial	Fecal energy percentage	Q1. Clear in study what is the cause and what is the effect?	Yes. Baseline measurements were obtained before antibiotic treatment and repeated after treatment.
			Q2. Control group?	No. No separate control group was included.
			Q3. Participants included in comparison similar?	Yes. The same participants were assessed before and after treatment.
			Q4. Participants included in comparison received similar care?	Yes. The same participants underwent the same study procedures before and after treatment.
			Q5. Multiple measurements of outcome (pre and post intervention)	No. One energy-utilization assessment was performed before and one after treatment.
			Q6. Outcomes of participants included in comparison measured the same way?	Yes. The same energy-utilization protocol was repeated before and after treatment.
			Q7. Outcomes measured in a reliable way?	Yes. Energy intake and fecal energy were assessed using a controlled 3-day diet, complete dye-delimited stool collection, and bomb calorimetry.
			Q8. Follow-up complete? If not, differences between groups (follow-up) adequately described and analyzed?	Yes. All 11 participants completed the study procedures and evaluations.
			Q9. Appropriate statistical analysis?	Yes. Paired analyses were used for before-after comparisons and descriptive fecal energy % was summarized using mean and standard deviation.
**Nielsen 1995**	Single arm trial	Fecal energy loss during refeeding per kg bodyweight (kJ/kg/day)	Q1. Clear in study what is the cause and what is the effect?	Yes. Nutritional measurements were obtained before and during/after increased oral nutritional intake.
			Q2. Control group?	No. No separate control group was included.
			Q3. Participants included in comparison similar?	Yes. The same participants were followed throughout the refeeding period.
			Q4. Participants included in comparison received similar care?	Yes. Patients followed the same structured refeeding and metabolic-balance protocol.
			Q5. Multiple measurements of outcome (pre and post intervention)	Yes. Nutritional intake and balance measurements were repeatedly assessed during the study period.
			Q6. Outcomes of participants included in comparison measured the same way?	Yes. The same metabolic-balance procedures were used throughout the follow-up.
			Q7. Outcomes measured in a reliable way?	Unclear. Energy balance measurements were performed objectively, but fecal-energy results were reported only for a five patient subset and were aggregated across repeated 3-day periods.

			Q8. Follow-up complete? If not, differences between groups (follow-up) adequately described and analyzed?	Yes. Study participation and duration were reported, with repeated measurements obtained during refeeding.
			Q9. Appropriate statistical analysis?	Yes. Repeated measurements and changes over time were analyzed using appropriate statistical methods.
**Pinar 2025**	Single arm trial	Absolute change in intestinal energy absorption	Q1. Clear in study what is the cause and what is the effect?	Yes. Baseline measurements preceded glepaglutide treatment and were repeated at week 24.
			Q2. Control group?	No. No separate control group was included.
			Q3. Participants included in comparison similar?	Yes. The same participants were assessed at baseline and week 24.
			Q4. Participants included in comparison received similar care?	Yes. The same metabolic-balance procedures were used at both assessments.
			Q5. Multiple measurements of outcome (pre and post intervention)	No. One baseline and one week-24 metabolic-balance assessment were performed.
			Q6. Outcomes of participants included in comparison measured the same way?	Yes. The same 48-hour metabolic-balance protocol was used at baseline and week 24.
			Q7. Outcomes measured in a reliable way?	Yes. Energy absorption was measured using 48-hour duplicate dietary and fecal collection with bomb calorimetry.
			Q8. Follow-up complete? If not, differences between groups (follow-up) adequately described and analyzed?	Yes. All 10 treated patients contributed to the week-24 metabolic-balance analysis.
			Q9. Appropriate statistical analysis?	Yes. Paired analyses were used for baseline-to-week-24 changes.

Methodological appraisal (n = 1)

This appraisal is not a validated risk-of-bias instrument. No established tool applies to a methodological transferability substudy, so the items below were adapted to describe the completeness of methodological reporting. The ratings are not risk-of-bias judgements and are not comparable with those obtained from the JBI checklists or from RoB 2; they are reported here for transparency only.

Study	Design	Review outcome assessed	Item	Judgement / justification
**Verbiest 2025**	Methodological transferability substudy, nested within a single-arm open-label phase 2 trial	Transferability and agreement of stool bomb calorimetry	Q1. Study objective clearly defined?	Yes. The study clearly aimed to assess transferability of full metabolic balance studies between two centers.
			Q2. Sample and study procedures adequately described?	Yes. Seven SBS patients underwent three 72-h metabolic-balance studies each, with standardized collection and processing procedures.
			Q3. Reference and comparison methods clearly described?	Yes. Copenhagen served as the reference laboratory and the same analytical methods were transferred to Leuven.
			Q4. Sample handling standardized?	Yes. Duplicate oral intake and fecal samples were homogenized, aliquoted, frozen and processed according to a predefined protocol.
			Q5. Bomb calorimetry methods adequately described?	Yes. Energy was measured using an IKA C6000 with predefined measurement conditions and quality-control procedures.
			Q6. Replicate measurements performed?	Yes. Each energy sample was measured in duplicate with predefined acceptance criteria.
			Q7. Quality control/calibration included?	Yes. Benzoic acid was measured in duplicate as a control before sample measurements.
			Q8. Agreement assessed appropriately?	Yes. Agreement was assessed using Bland-Altman analysis, coefficient of variation and intraclass correlation coefficients.
			Q9. Potential methodological limitation acknowledged?	Yes. The authors acknowledged remaining uncertainty regarding homogenization quality and the need for further validation.

RoB 2 (n = 1)

Study	Design	Review outcome assessed	Item	Judgement / justification
**Naimi 2019**	Randomized cross-over study	Change from baseline in absolute energy absorption (kJ/day)	D1. Bias arising from the randomization process	Low risk. Treatment sequences were computer-generated by an independent statistician and allocation was concealed.
			D2. Bias due to deviations from intended interventions	Some concerns. The trial was double-blind, but efficacy analyses were performed in the per-protocol population rather than all randomized participants
			D3. Bias due to missing outcome data	Some concerns. Two randomized participants did not complete both treatment periods and were excluded from the per-protocol efficacy analysis.
			D4. Bias in measurement of the outcome	Low risk. Metabolic balance outcomes were objectively measured and treatment allocation was masked.
			D5. Bias in selection of the reported result	Low risk. Outcomes and statistical analyses were prespecified and the trial was registered.
			Overall	Some concerns. Not included in meta-analysis as there is no baseline data available

Supplementary Table S4. Reports not retrieved

Following the revised search of 28 August 2026, two reports could not be obtained because no full-text publication was available.

Arm	Report	Reason not retrieved	Relevance
Databases and registers	Fernandes J. General aspects of nutrition, absorption and metabolism in patients with cystic fibrosis, 1985.	No full text obtainable	Population outside the scope of this review.
Citation searching	Woolf GM, Miller C, Kurian R, Jeejeebhoy KN. Nutritional absorption in short bowel syndrome: evaluation of fluid, calorie, and divalent cation requirements. *Dig Dis Sci* 1987;32:8–15.	No full text obtainable	Reports intestinal energy absorption measured by bomb calorimetry in adults with short bowel syndrome and would, on the information available, have met the eligibility criteria. Distinct from Woolf 1983, which was retrieved and is included.

PRISMA reconciliation

Arm	Reports sought	Not retrieved	Assessed	Excluded	Included
Databases and registers	47	1	46	24	22
Citation searching	23	1	22	16	6
**Total**	**70**	**2**	**68**	**40**	**28**

Note on reports recorded as unretrieved in an earlier version

Several reports recorded as unretrieved during the initial review were subsequently obtained and assessed. Wierdsma et al. (2005), published in Dutch and not indexed under the English title by which it had been identified, was obtained and excluded at full-text assessment as a two-patient case report, an ineligible study design. Woolf (1983), Nielsen (1995) and others were obtained and are included in the review.

Supplementary Figure S1. Risk-of-bias appraisal of the 28 included studies. Judgements are shown per item for each instrument: the JBI Critical Appraisal Checklist for Analytical Cross-Sectional Studies (21 studies), the JBI Critical Appraisal Checklist for Quasi-Experimental Studies (5 studies) and the Cochrane Risk of Bias 2 tool (1 study). The fourth panel shows an adapted methodological appraisal of one methodological transferability substudy, for which no validated risk-of-bias instrument applies; it is shown in grey because it is not a risk-of-bias assessment and its ratings are not comparable with those from the JBI checklists or RoB 2. Symbols denote the judgement independently of colour: + yes / low risk, – no / high risk, ? unclear or some concerns, and blank not applicable to the outcome assessed in this review. Item-level justifications are given in Supplementary Table S3.

JBI Critical Appraisal Checklist for Analytical Cross-Sectional Studies (n = 21)

	Q1	Q2	Q3	Q4	Q5	Q6	Q7	Q8
Heymsfield 1981	?	?		+			+	+
Woolf 1983	+	+		+			+	+
Messing 1991	+	+		+			+	+
Nordgaard 1994	+	+		+			+	+
Jeppesen 1998	+	+		+			+	+
Heydorn 1999	+	+		+			+	+
Jeppesen 2000	+	+		+			+	+
Haderslev 2003	+	+		+			+	+
Strack van Schijndel 2006	+	+		?			+	+
Vaisman 2006	+	+		?			+	+
Achour 2007	?	+		?			+	+
Peters 2007	+	+		?			+	+
Joly 2009	+	+		+			+	+
Wierdsma 2011	+	+		?			+	+
Wierdsma 2016	+	+		?			+	+
Witvliet-van Nierop 2017	?	+		?			+	+
Erchinger 2018	+	+		?			+	+
Brownell 2020	+	+		?			+	+
Qian 2024	?	+		?			+	+
Verbiest 2024	+	+		+			+	+
Andreae 2026	?	+		?			+	+

JBI Critical Appraisal Checklist for Quasi-Experimental Studies (n = 5)

	Q1	Q2	Q3	Q4	Q5	Q6	Q7	Q8	Q9
Nielsen 1995	+	−	+	+	+	+	?	+	+
Atia 2011	+	−	+	+	−	+	+	+	+
Mathur 2016	+	−	+	+	−	+	+	+	+
Eliasson 2022	+	−	+	+	−	+	+	+	+
Pinar 2025	+	−	+	+	−	+	+	+	+

Cochrane RoB 2 (n = 1)

	D1	D2	D3	D4	D5	Overall
Naimi 2019	+	?	?	+	+	?

Adapted methodological appraisal (n = 1)

	Q1	Q2	Q3	Q4	Q5	Q6	Q7	Q8	Q9
Verbiest 2025	+	+	+	+	+	+	+	+	+

Not a validated risk-of-bias instrument: these ratings describe completeness of methodological reporting and are not comparable with the JBI or RoB 2 judgements (shown in grey for that reason).

Item wording is given in Supplementary Table S3. Each study was appraised against the specific outcome used in this review (intestinal energy absorption or apparent energy digestibility).

Yes   Unclear / some concerns   No   Not applicable
